# Design of aqueous redox-enhanced electrochemical capacitors with high specific energies and slow self-discharge

**DOI:** 10.1038/ncomms8818

**Published:** 2015-08-04

**Authors:** Sang-Eun Chun, Brian Evanko, Xingfeng Wang, David Vonlanthen, Xiulei Ji, Galen D. Stucky, Shannon W. Boettcher

**Affiliations:** 1 Department of Chemistry and Biochemistry, University of Oregon, Eugene, Oregon 97403, USA; 2 Materials Department, University of California, Santa Barbara, California 93106, USA; 3 Department of Chemistry, Oregon State University, Corvallis, Oregon, 97331, USA; 4 Department of Physics, University of California, Santa Barbara, California 93106, USA; 5 Department of Chemistry and Biochemistry, University of California, Santa Barbara, California 93106, USA

## Abstract

Electrochemical double-layer capacitors exhibit high power and long cycle life but have low specific energy compared with batteries, limiting applications. Redox-enhanced capacitors increase specific energy by using redox-active electrolytes that are oxidized at the positive electrode and reduced at the negative electrode during charging. Here we report characteristics of several redox electrolytes to illustrate operational/self-discharge mechanisms and the design rules for high performance. We discover a methyl viologen (MV)/bromide electrolyte that delivers a high specific energy of ∼14 Wh kg^−1^ based on the mass of electrodes and electrolyte, without the use of an ion-selective membrane separator. Substituting heptyl viologen for MV increases stability, with no degradation over 20,000 cycles. Self-discharge is low, due to adsorption of the redox couples in the charged state to the activated carbon, and comparable to cells with inert electrolyte. An electrochemical model reproduces experiments and predicts that 30–50 Wh kg^−1^ is possible with optimization.

## Abstract (web summary)

The energy density of electrochemical capacitors can be increased by using a redox-active electrolyte, but such capacitors often suffer from significant self-discharge and low operating voltage. Here, the authors report a new redox-active aqueous electrolyte to effectively tackle the problems.

## Graphical abstract

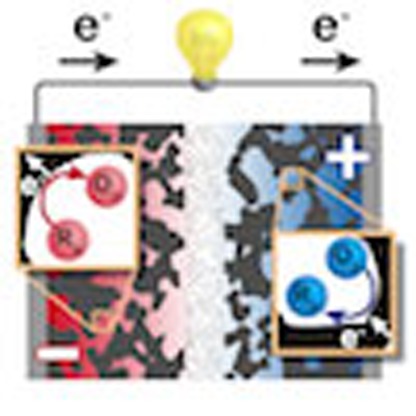

Electrochemical double-layer capacitors (EDLCs) store electrical energy at the interface between a solid electrode (for example, high-surface-area-activated carbon) and a liquid electrolyte^1^,^2^,^3^,^4^. They are used in commercial applications requiring high power density and long-term cycle stability, for example, in load-leveling and in electric vehicles^5^,^6^. These characteristics are enabled by a double-layer charging mechanism, which relies on physical ion adsorption/desorption in the Helmholtz layer of the liquid electrolyte and does not require slower solid-state ion-insertion/de-insertion reactions as in, for example, Li-ion batteries^1^,^7^,^8^, which also lead to electrode volume change and thus capacity fade with cycling^9^,^10^,^11^. To attain specific energies of 5–10 Wh kg^−1^, commercial EDLCs require organic electrolytes that operate at high potentials near 3 V. The disadvantages of these electrolytes are (1) low-to-moderate volumetric and gravimetric energy density, (2) high cost, (3) the requirement for high-purity-activated carbon (needed to reduce self-discharge at the high voltages)^12^ and (4) safety concerns related to flammability^4^. These disadvantages limit applications of EDLCs^1^,^5^.

One challenge in increasing the energy density of EDLCs is the mass of the electrolyte^13^,^14^. High-surface-area carbons typically have large pore volumes that fill with inert electrolyte, reducing the cell-level-specific energy^13^. A number of hybrid and pseudocapacitive devices incorporate solid-state or surface-redox functionality into electrodes to increase the specific energy^15^,^16^,^17^,^18^.

In ‘redox EDLCs' the inert electrolyte is replaced with a redox-active one, thereby adding faradaic charge-storage mechanisms to the underlying capacitive ones while ideally maintaining high power and cyclability (Fig. 1)^19^,^20^,^21^,^22^. This approach enables the use of aqueous (aq.) electrolytes (where high redox couple solubility results in high capacity) and less-expensive carbons (due to lower operating voltage windows). The disadvantages include the possibility of internal self-discharge via shuttling of mobile redox species, diffusion overvoltage losses and cycling instability due to the intermixing redox couples^20^,^22^,^23^. We show that these challenges can be simultaneously mitigated after understanding the fundamental electrochemical processes.

A number of couples have been studied in redox EDLCs including halides, vanadium complexes, copper salts, methylene blue, phenylenediamine, indigo carmine and quinones^19^,^20^,^22^,^24^,^25^. The performance to date has been low (see Supplementary Table 1 for a comparison of reported work). The most substantial work is by Frackowiak and co-workers^4^,^21^, who developed aq. potassium iodide (KI) and VOSO_4_ ‘catholyte' and ‘anolyte', respectively, separated by a Nafion membrane in two cell compartments. Although specific energy >20 Wh kg^−1^ and specific power >2,000 W kg^−1^ were reported^21^, these metrics are normalized to the mass of the electrodes alone^13^,^26^,^27^. While such normalization is common, it is inappropriate for redox EDLCs, where the electrolyte contributes directly to faradaic storage. Accounting for electrolyte (see discussion) reduces performance metrics by at least a factor of 3. Further, the cost of the Nafion cation-exchange membrane, which is used to prevent self-discharge via redox shuttling, is prohibitive^4^,^21^,^23^. Stucky and co-workers studied^25^ a related system containing KI/VOSO_4_ electrolyte without an ion-selective separator. They proposed an electrostatic mechanism to account for self-discharge times in the order of 1 h, which was somewhat longer than expected given the separator used.

More-substantial progress has been prevented by the demands on the redox couples needed for the electrolyte. (1) The couples must be soluble at high concentrations, ideally >1 M, to contribute substantially to the capacity. (2) The electron-transfer kinetics must be sufficiently fast to minimize voltage loss during charge/discharge. (3) The solution behaviour of the charged redox couples must enable slow self-discharge, without the use of expensive ion-selective membranes. (4) During charging, one couple (O_p_/R_p_) must be oxidized at the positive electrode while the other (O_n_/R_n_) must be reduced at the negative electrode. The relevant redox potentials of the two species should be near the electrolyte decomposition window to maximize the voltage. In water at near-neutral pH, appropriate couples could span >1.5 V as the water oxidation and reduction kinetics are slow relative to those of one-electron couples^28^.

Here we report record performance for redox EDLCs using a new electrolyte containing viologen and bromide that was optimized with respect to the above criteria by systematic study of the underlying electrochemical phenomena. A device with 0.4 M potassium bromide (KBr) and 0.1 M heptyl viologen (HV) provides a specific energy of 11 Wh kg^−1^ at a specific power of 122 W kg^−1^ (normalized to the total mass of both electrodes and electrolyte, with each electrode having a high mass loading of 12.9 mg cm^−2^), and shows negligible fade over 20,000 cycles and slower self-discharge than aq. control EDLCs without redox-active electrolyte. Using methyl viologen (MV) instead of HV provides higher energy density (∼14 Wh kg^−1^) due to higher solubility, but also faster self-discharge. The self-discharge mechanisms are studied by comparing the behaviour of redox electrolytes with varying chemical structure and molecular charge. Adsorption of the viologen and Br redox species to the activated carbon following charging is found to be important, and this effect enables 90% retention of stored energy after 6 h at open circuit. In addition to good electrochemical performance, the cells that use aq. electrolyte are simple to prepare under ambient conditions and are likely to be safer for application than those using flammable organics^4^,^16^. The new system is thus appealing as a low-cost aq. capacitor/battery hybrid.

## Results

### Cell design and performance analysis

To quantitatively analyse charge–discharge and performance data, two custom cells were designed and built (see Methods section and Supplementary Fig. 1). The first was a T-shaped Swagelok cell that incorporated a reference electrode with the tip placed at the edge of the separator (Supplementary Fig. 1a). This enabled the study of the electrochemical processes at both electrodes simultaneously. Insulated glassy carbon (GC) current collectors were used to avoid background current that might be associated with metallic current collectors. These Swagelok-type cells were flooded with excess electrolyte (∼500 μl) and thus the specific energy and power were calculated based on the combined ‘dry' electrode mass (indicated as g_dry_) from the discharge data (as is typical and discussed below)^29^,^30^.

We also designed a two-electrode cell with a ‘volume-limiting' geometry, where the electrolyte is confined within a machined cavity and sandwiched between two GC plates in contact with the activated carbon (Supplementary Fig. 1b). For the volume-limiting cell, the mass of both electrodes and all electrolyte were used to calculate the specific energy, power and capacity (indicated as g_wet_). The polycarbonate separator mass (0.9 mg) was ignored as it is only ∼1% of the total cell mass. Performance metrics based on the total ‘wet' cell mass are practically relevant, whereas the ‘dry' metrics are useful only for comparison to other systems where electrolyte mass has been ignored (Supplementary Table 1).

These well-defined cell geometries enabled rigorous definition and evaluation of performance metrics. The specific capacity at discharge, *Q*_dis_ (mAh g^−1^), is defined as


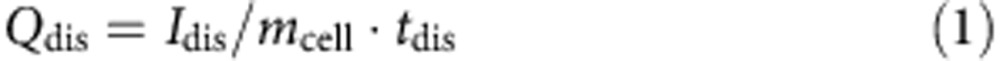


where *I*_dis_ is the constant discharge current in mA, *m*_cell_ is the mass of the cell components (as indicated above either ‘dry' or ‘wet') and *t*_dis_ is the discharge time. The specific energy density at discharge, *E*_dis_ (Wh kg^−1^), is calculated by integrating the instantaneous specific power


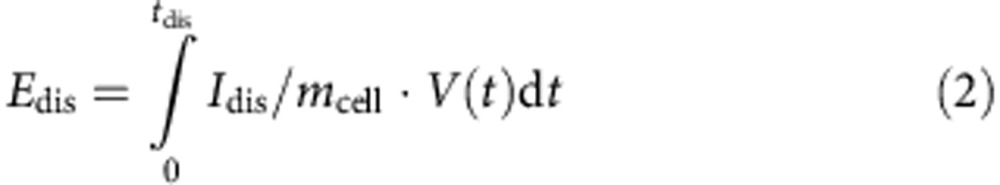


where *V*(*t*) is the time-varying voltage output of the cell. The average specific power, *P*_dis_ (W kg^−1^), is calculated by dividing the energy by the discharge time^14^,^30^,^31^


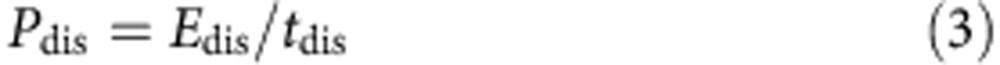


We define the coulombic efficiency, *η*_C_(*δ*), after a specific self-discharge period *δ* (in min, where the cell is left at open circuit) as


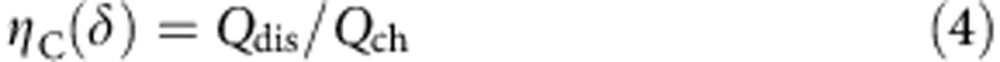


where *Q*_ch_ is the total charge passed during the charging cycle (analogous to *Q*_dis_ defined in equation (1)). We define the energy efficiency *η*_E_(*δ)* after a specific self-discharge time as


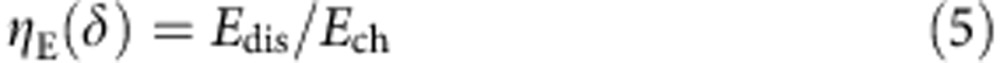


where *E*_ch_ is the total energy expended during the charging cycle (analogous to *E*_dis_ defined in equation (2)). Finally, we define the energy retention efficiency, *η*_R_(*δ)*, which is the ratio of the energy obtained after a self-discharge period *δ* compared with that where *δ*=0, as


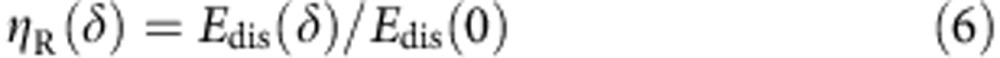


Self-discharge was studied by measuring *η*_C_ and *η*_E_ as a function of *δ* (see Supplementary Fig. 2 and Supplementary Note 1 for details). The cell was first charged to the voltage indicated at 0.5 A g_dry_^−1^. We use the ‘half-life' of the specific energy 
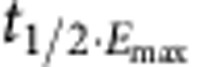
, where 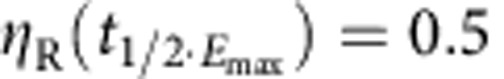 as well as *η*_R_(6 h) to compare the different self-discharge rates of the cells. When 
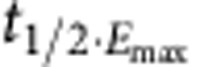
 was longer than the test duration (6 h), the experimental *η*_R_ data was fit to a linear decay to estimate 
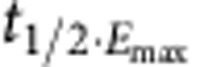
.

### Redox-active electrolytes for the positive electrode

Redox species at the positive electrode are reduced during discharge (O_p_+e^−^→R_p_) and thus referred to as the ‘catholyte'. The catholyte should have a reduction potential near, or slightly more positive than, the oxygen evolution potential to maximize energy density. The halogens iodide and bromide are promising as they are inexpensive and highly soluble (>1 M)^32^,^33^. The aq. reduction potential of iodine (
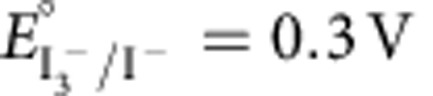
 versus the standard calomel electrode (SCE)) is within the water stability window, while that of bromine (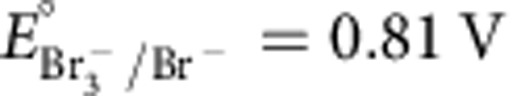 versus SCE) is above the thermodynamic oxygen evolution potential at pH 7 (0.58 V versus SCE)^34^.

**Figure 2:**
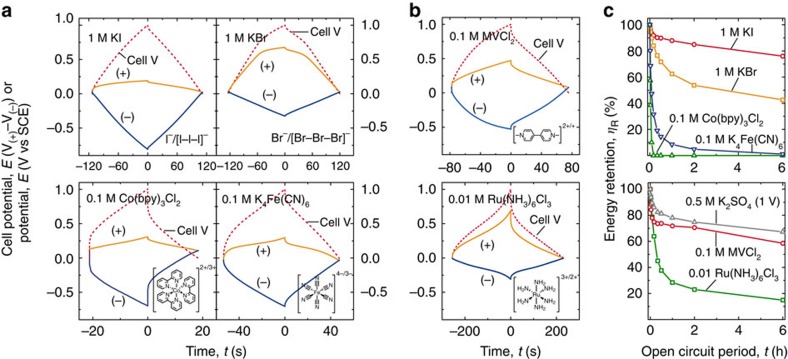
Electrochemical characterization of cells with a single redox couple. The galvanostatic charge/discharge profiles (dashed red lines) of the candidate redox couples are shown for (**a**) the positive electrode (solid orange) and (**b**) the negative electrode (solid blue), respectively, referenced to the centrally placed SCE. The cells were charged/discharged at a rate of 1 A g^−1^_(+) electrode_ (normalized to the mass of the positive electrode only, because the negative electrode mass was varied to accommodate couples with different redox potentials) to a total cell voltage of 1 V. (**c**) Energy retention *η*_R_ for the cells in **a**,**b**. Each data point was collected by charging the cell, allowing it to sit at open circuit for a given time, then discharging the cell completely. In **a**, the slope of the potential–time curve for the negative electrode is smaller for the KBr cell than for the KI cell, because the negative electrode had three times the mass of the positive electrode so that the positive electrode was able to reach the Br^−^ oxidation potential with a total cell potential of 1 V, which was not possible with a symmetric cell (as in Supplementary Fig. 3).

Figure 2a shows the galvanostatic cycling profiles of candidate catholytes tested in a three-electrode Swagelok cell. For 1 M KI, the positive electrode potential narrowly varied between 0.02 V and 0.19 V versus SCE, indicating oxidation of I^−^ to I_3_^−^ (the difference between the observed plateau potential and 
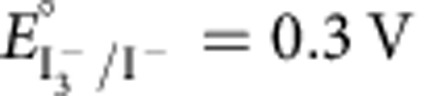
 versus SCE is discussed below)^34^. The negative electrode potential varied linearly with charge between 0.02 V and −0.81 V versus SCE, indicating a double-layer charging mechanism with inert K^+^.

For 1 M KBr electrolyte, the positive electrode shows two distinct charging regimes. For the first 60 s, the electrode potential depends linearly on the charge, indicating capacitive charging with Br^−^ in the double layer. For the next 60 s, the potential increase slows and plateaus at ∼0.7 V versus SCE, indicating oxidation of Br^−^ to Br_3_^−^ (ref. 34). The negative electrode shows purely capacitive charging. The high redox potential of Br^−^/Br_3_^−^ is advantageous for increasing energy density.

EDLC self-discharge is commonly studied by monitoring the potential decay at open circuit^35^,^36^. However, because the charge is not linear with potential for redox EDLCs, we measured energy retention *η*_R_ by complete discharge at each time point in Fig. 2c. The self-discharge profiles of KI and KBr show *η*_R_ of 76% and 43% after 6 h, respectively (Fig. 2c). Remarkably, the self-discharge rate of the KI cell is slower than that of the control potassium sulfate (K_2_SO_4_) cell when also charged to 1 V (*η*_R_=67% after 6 h). Given the lack of an ion-selective membrane, the slow self-discharge of the halogen cells is unexpected. After charging the KI cell, a large concentration gradient of 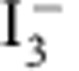 and I^−^ between the positive and negative electrode is present that would normally be expected to drive diffusive transport across the cell resulting in fast self-discharge.

One hypothesis that might explain the remarkably slow self-discharge is that the negatively charged oxidation products 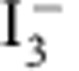 or 
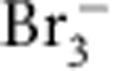
 are electrostatically held in the double layer of the positively charged activated carbon electrode. To test this, we fabricated new cells with potassium ferrocyanide (K_4_Fe(CN)_6_) and tris(2,2′-bipyridyl)dichloro-cobalt (Co(bpy)_3_Cl_2_) redox-active electrolytes. Both couples have reduction potentials similar to 
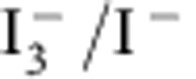
 and fast kinetics (Supplementary Figs 4 and 5). Co(bpy)_3_^2+/3+^ has a positive charge, and thus would be expected to be expelled from the double layer at the positive electrode (where it is oxidized) and subsequently reduced after diffusing to the negative electrode, thus increasing the self-discharge rate. Fe(CN)_6_^4−/3−^ has a negative charge, like I_3_^−^/I^−^, and thus might also show retarded self-discharge if electrostatic effects play the dominate role.

K_4_Fe(CN)_6_ and Co(bpy)_3_Cl_2_ cells show galvanostatic charging behaviour at the positive electrode similar to the KI cell with the electrode potential pinned near the standard potential of the couple (Fig. 2a). The negative electrodes show capacitive (linear potential–time) response on charging. On discharging, an additional potential loss associated with the low ionic conductivity of the 0.1 M redox-active electrolytes is measured. The Co(bpy)_3_Cl_2_ and K_4_Fe(CN)_6_ cells have 89.5% and 90.8% coulombic efficiency, respectively—lower than the 99.9% and 98.8% measured for the KI and KBr cells, respectively. The Co(bpy)_3_Cl_2_ cell loses half of its energy in 1 min, while for the K_4_Fe(CN)_6_ cell this takes 5 min (Fig. 2c). This data is consistent with electrostatics contributing to the self-discharge in the case of Co(bpy)_3_^2+/3+^ and retarding it in the case of Fe(CN)_6_^4−/3−^, and for the halides. However, despite the large negative charge of Fe(CN)_6_^4−/3−^, its self-discharge rate is still roughly 100 times that of 
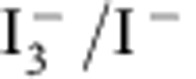
. The retarded self-discharge for the halides cannot be explained purely by electrostatics.

Another mechanism to explain the slow self-discharge is physical adsorption of the oxidized species within the activated carbon surface preventing cross diffusion. Halides are known to adsorb on carbon electrodes^37^,^38^,^39^ and we confirmed the strong adsorption of Br_3_^−^ using ultraviolet–visible spectroscopy. In contrast, Br^−^, Fe(CN)_6_^4−^ and Fe(CN)_6_^3−^ do not adsorb (see Supplementary Note 2 and Supplementary Fig. 6). Further, the observed potential plateau for both the KBr and KI cells (Fig. 2a) is ∼0.1 V less positive than the standard potential of the respective couples. This is consistent with specific adsorption stabilizing the oxidized halide, shifting the formal potential within the activated carbon negative of the standard potential. These results show that physical adsorption is the primary mechanism preventing self-discharge and electrostatic effects are secondary.

### Redox-active electrolytes for the negative electrode

Redox couples for the negative electrode (O_n_/R_n_) should have standard potentials near the hydrogen evolution potential, as well as high solubility and solution compatibility with the catholyte. Based on the findings discussed above, O_n_/R_n_ should have a positive charge and physically adsorb on activated carbon following charging to prevent self-discharge.

Viologen dications (4,4′-bipyridinium compounds) are positively charged, highly soluble redox couples, with formal potentials negative of the hydrogen potential and fast, reversible, kinetics^40^,^41^,^42^,^43^,^44^,^45^. MVCl_2_ (1,1′-Dimethyl-4,4′-bipyridinium dichloride) was studied first due to its negative potential (*E*^o^=−0.69 V versus SCE), commercial availability and low cost (<$5 kg^−1^ in bulk, used for agriculture)^43^,^46^,^47^. Being a nearly co-planar *π–π* conjugated ring system, MVCl_2_ also adsorbs on activated carbon surfaces^48^. Stronger adsorption is expected after reduction of MV^2+^ to MV^+^ due to decreased charge density and increased co-planarity of the two adjacent rings.

**Figure 3:**
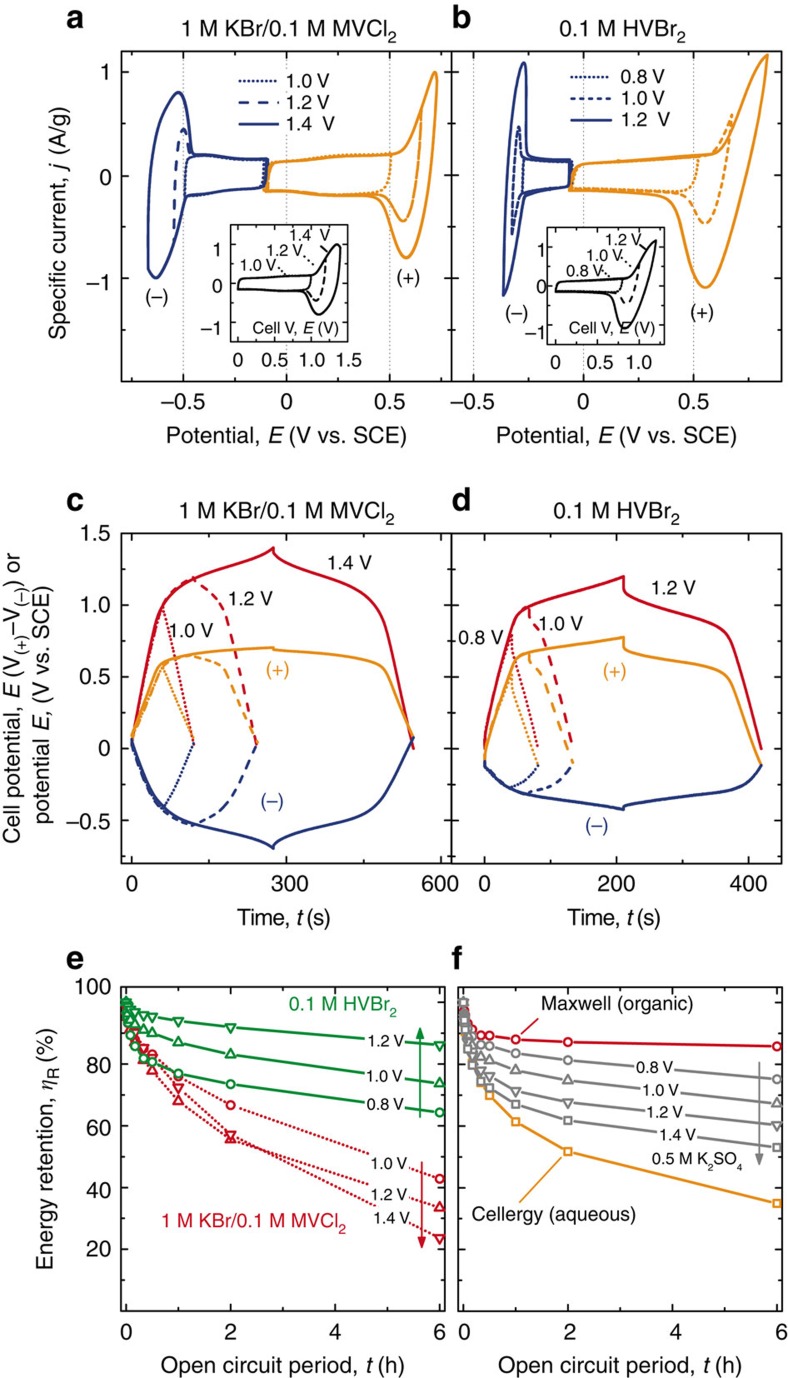
Electrochemical characterization of cells with combined electrolytes. **a**,**b** show voltammograms for the complete cell collected at 5 mV s^−1^ (inset) with the voltages for the positive (orange) and negative (blue) electrodes separately recorded. **c**,**d** show the galvanostatic charge/discharge profiles at 0.5 A g_dry_^−1^ to a total cell potential between 0.8 and 1.4 V. **e** shows the energy retention *η*_R_ for the cells with redox-active electrolyte, demonstrating substantial differences in the self-discharge rate between cells with HV and those with MV. **f** shows *η*_R_ for the control cells tested with inert electrolyte and commercial EDLCs (See Supplementary Note 3, Supplementary Fig. 11 and Supplementary Table 2).

To study the viologen electrochemistry in the absence of a redox-active electrolyte at the positive electrode, a 4:1 mass ratio for the positive/negative electrode was used. During galvanostatic charging, the positive electrode potential varies nearly linearly with time, while the negative electrode potential curves with time/charge near ∼−0.5 V versus SCE (Fig. 2b), consistent with the reduction of MV^2+^ to MV^+^ (Supplementary Fig. 7). The self-discharge rate of the MVCl_2_ electrolyte was measured (Fig. 3c) and found comparable to that of the EDLC with 0.5 M K_2_SO_4_, suggesting that MV^2+^/MV^+^ does not contribute substantially to self-discharge via redox shuttling.

To understand the self-discharge processes, a ruthenium hexamine dichloride electrolyte was also studied (Fig. 2b). Ru(NH_3_)_6_^3+^/Ru(NH_3_)_6_^2+^ is cationic, like MV^2+^/MV^+^, but is unlikely to specifically adsorb on the activated carbon surface because of its near-spherical molecular shape. The hexaammineruthenium (III) chloride (Ru(NH_3_)_6_Cl_3_) cell shows double-layer charging with Cl^−^ on the positive electrode and some faradaic charging on the negative electrode with a slight plateau near −0.2 V consistent with the reduction of Ru(NH_3_)_6_^3+^ (*E*^o^∼−0.14 V versus SCE, Supplementary Fig. 8)^49^. The self-discharge rate (Fig. 2c), however, is significantly faster for Ru(NH_3_)_6_Cl_3_ than for MVCl_2_, providing further evidence that electrostatic effects are not sufficient to prevent self-discharge and that MV^+^, like the oxidized halides, adsorbs on the carbon electrode^50^. Ultraviolet–visible spectroscopy supports this mechanism, showing weak adsorption of MV^2+^ and strong adsorption of MV^+^ (see Supplementary Note 2 and Supplementary Fig. 6).

### Combined electrolyte systems

For high performance, the redox-active electrolytes for the positive and negative electrodes must be combined. All species in both redox states (O_n_, R_n_, O_p_ and R_p_) should be stable in the same electrolyte to reduce manufacturing complexity and demands on the separator. We first studied MVCl_2_/KI, which was stable in the uncharged state but formed MV ^+^-I^−^ precipitate on charging (Supplementary Fig. 9) leading to irreversible capacity loss^41^,^51^. In contrast, MVCl_2_/KBr solutions showed highly reversible redox behaviour of both redox ions in analytical voltammetry cells (Supplementary Fig. 10). Br^−^ benefits from a more-positive oxidation potential compared with I^−^, thus providing increased specific energy, but is more reactive/corrosive.

Cells with 1 M KBr/0.1 M MVCl_2_ electrolyte were tested by voltammetry and galvanostatic cycling to probe the charging mechanism (Fig. 3a,c). Voltammograms collected with a 1 V window are rectangular, indicating capacitive charging at both electrodes. As the Br/MV cell was cycled beyond 1 V, current peaks were observed at the positive and negative electrodes corresponding to Br^−^ oxidation to Br_3_^−^ and MV^2+^ reduction to MV^+^, respectively, (Fig. 3a).

Figure 3c shows the galvanostatic charging of the same cells to different total cell voltages. At both the positive and negative electrodes (orange and blue traces, respectively) an initial capacitive (linear) voltage–time response is observed followed by a faradaic response as the electrode potential approaches the reduction potential of the couple.

Figure 3e,f shows that the self-discharge rate for the 1 M KBr/0.1 M MVCl_2_ cell charged to 1.4 V 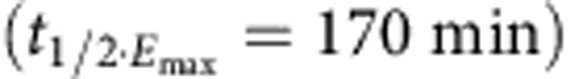 is similar to the commercial Cellergy capacitor 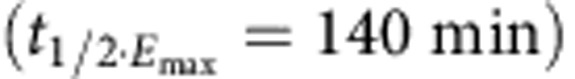, but still faster than the commercial non-aq. Maxwell cell and the KI or KBr aq. cells 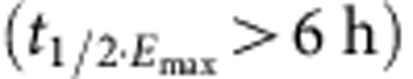, suggesting that MV^2+^/MV^+^ may contribute to self-discharge under these conditions.

To improve the self-discharge (and cycle stability as discussed below), we explored different viologens. HV forms a strongly bonded solid on electrodes following its reduction in aq. solution in the presence of Br^−^ or I^−^, as evidenced by voltammetry and ultraviolet–visible spectroscopy^42^,^43^,^52^,^53^,^54^,^55^. This adsorption/insolubility could be useful to impede internal shunting as long as the adsorption process is reversible. We tested HVBr_2_ (1,1′-Diheptyl-4,4′-bipyridinium dibromide; the chloride salt is not commercially available) in cells in an identical manner as described above for the KBr/MVCl_2_ cells. The results are similar with the exception that HV^2+^ has a more-positive reduction potential (*E*^o^=−0.60 V versus SCE) than MV^2+^ leading to a lower plateau voltage^41^,^43^. The galvanostatic charge–discharge curves (Fig. 3d) also show evidence of higher ionic resistance due to lower electrolyte concentration.

The self-discharge rate of the HVBr_2_ electrolyte is substantially lower than that for KBr/MVCl_2_. Further, the self-discharge rate for HVBr_2_ decreases as the charging voltage is increased, whereas it increases for KBr/MVCl_2_ with voltage. This data is consistent with the strong adsorption/precipitation of the HV^+^ onto the activated carbon following charging (Supplementary Fig. 10). The weaker-adsorbing MV^+^ apparently contributes to self-discharge when large concentrations accumulate at high states of charge. The formation of HV^+^ films was confirmed by voltammetry and visual observation on a GC electrode in an analytical half-cell (Supplementary Fig. 10). In addition, the thin HVBr_2_ layer formed on the electrode might block impurity transport, which provides a significant self-discharge path in aq. EDLCs with inert electrolyte^35^. The adsorption/precipitation process was completely reversible with the HVBr_2_ showing nearly 100% coulombic efficiency. The concept of using an immobilized redox-ion on the electrode surface to limit self-discharge was proposed recently using the Cu^2+^/Cu^0^ couple, but this requires acidic conditions, is susceptible to dendrite formation, and no complementary couple was used at the opposite electrode^23^.

Table 1 shows the specific capacity and energy of the redox-enhanced cells evaluated with galvanostatic cycling. These data were collected in the Swagelok cell with excess electrolyte and therefore the performance metrics were normalized to the mass of the electrodes only and do not include the electrolyte (as is typical). The dramatic effect of the added faradaic reaction is apparent; the 1 M KBr/0.1 M MVCl_2_ cell at 1.4 V (driving faradaic+capacitive charging) stores over four times more charge than at 1.0 V (capacitive charging only), and has eight times larger specific energy.

### Performance quantification and optimization

To increase energy density and minimize ionic resistance, higher redox-active electrolyte concentrations are needed. Further, the practical specific energy and power must be evaluated based on both electrode and electrolyte mass^13^. MVCl_2_ is highly soluble in water (>2 M), whereas the HVBr_2_ solubility is <0.2 M (ref. 56). 1.0 M KBr/0.5 M MVCl_2_ and 0.4 M KBr/0.1 M HVBr_2_ were tested in the two-electrode ‘volume-limiting' cell, which accommodates a precise quantity of electrolyte. Thin polycarbonate film (9 μm) instead of Whatman paper (180 μm) was used as a separator to minimize excess electrolyte. The KBr/HVBr_2_ cell with a polycarbonate separator exhibits 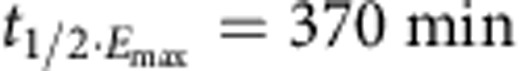, significantly faster self-discharge than with Whatman paper (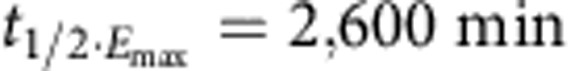, Supplementary Fig. 12), which is expected since the thinner separator provides a shorter diffusion pathway. Nonetheless, the self-discharge in the Br/HV cells is slower than control EDLCs using the same polycarbonate film 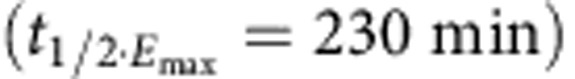. Further, the self-discharge rate decreased after prolonged cycling for HVBr_2_. After 1,000 galvanostatic charge/discharge cycles, *η*_R_(6 h)=87% and 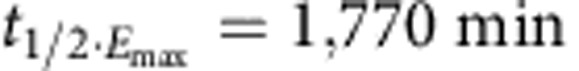. After 2,000 cycles, it increased to *η*_R_(6 h)=93% and 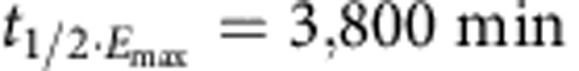 (Supplementary Fig. 13). This excellent self-discharge rate is an important figure of merit as a practical energy storage device.

Performance parameters measured from galvanostatic cycling at 0.5 A g_dry_^−1^ in volume-limiting cells are given in Table 2. The mass-based metrics are reported normalized to both the total active-material mass (electrodes+electrolyte) and, in parentheses, the electrode mass only. The specific energies achieved for the KBr/MVCl_2_ system (∼14 Wh kg_wet_^−1^ or 51 Wh kg_dry_^−1^, 16.8 mWh cm^−3^) and KBr/HVBr_2_ system (∼11 Wh kg_wet_^−1^ or 39 Wh kg_dry_^−1^, 12.7 mWh cm^−3^) are substantially larger than all previous reports of aq. redox EDLCs (see Supplementary Table 1). A concentrated MV^2+^ cell (1 M KBr/1 M MVCl_2_) was assembled to increase the energy density, but the cell showed lower coulombic efficiency with *η*_C_(0)∼90% (Supplementary Fig. 14).

**Figure 4:**
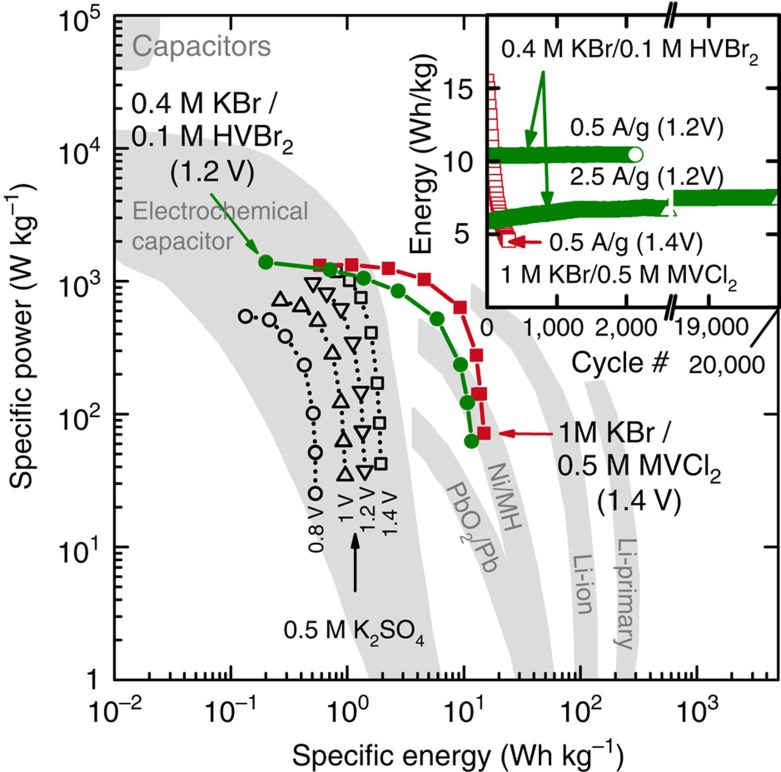
Ragone plot of redox-EDLC performance from volume-limiting cells. Data for 1 M KBr/0.5 M MVCl_2_ and 0.4 M KBr/0.1 M HVBr_2_ electrolytes are shown in red and green, respectively, and are overlaid on the performance of various electrochemical devices from ref. 2. The redox-EDLC performance metrics were normalized to the mass of both electrodes and electrolyte. The inset shows the long-term cycling performance of both cells obtained by galvanostatic cycling at 0.5 and 2.5 A g_dry_^−1^ (every tenth cycle marked). Higher current was used to speed up the 20,000 cycle test and the HV/Br cell showed no degradation at 0.5 A g_dry_^−1^ after the 20,000 cycle test at 2.5 A g_dry_^−1^.

We measured the relationship between energy and power, and compared the performance to control aq. K_2_SO_4_ devices and published reference data on a Ragone plot (Fig. 4 and Supplementary Fig. 15). The metrics estimated for both devices are promising as they fill a gap between high-power batteries and traditional EDLCs using simple, potentially low-cost, aq. chemistry. Additional optimization and cell engineering (discussed below) would further improve both power (reduced internal resistance) and specific energy.

To study degradation/fading, cells were cycled at 0.5 and 2.5 A g_dry_^−1^ (Fig. 4 inset). The energy density of the KBr/MVCl_2_ cell fades over the course of several hundred cycles. This degradation is likely due to irreversible polymerization of MV^+^ in the aq. electrolyte^41^,^43^. In comparison, the KBr/HVBr_2_ cell shows no fading over 2,000 cycles (0.5 A g_dry_^−1^) and 20,000 cycles (2.5 A g_dry_^−1^). The specific energy and power are largely limited by the lower solubility of HV^2+^. There are hundreds of viologen derivatives that can be synthesized—an appropriate combination of solubility and stability can likely be found^43^,^57^,^58^.

## Discussion

There has been previous work aimed at enhancing conventional EDLCs using faradaic charging from soluble redox species (see Supplementary Table 1). Previous cells often rely on a single redox process at one electrode coupled with capacitive charging at the other, but this capacity mismatch lowers cell-level performance^20^,^22^,^59^,^60^,^61^. Balancing the capacity with different redox species at each electrode has been demonstrated, but the potential difference between the couples has been small, leading to low specific energy when electrolyte weight is considered, and the cells have required ion-selective Nafion separators^21^. The Br/viologen cell in this work was designed and optimized to achieve high energy density considering all active components (electrode and electrolyte) and simultaneously shows better specific energy and self-discharge behaviour than previous redox-enhanced EDLCs.

It is also useful to compare these redox EDLCs to flow batteries^37^,^62^. While both store charge through oxidation and reduction of soluble couples, the underlying design principles and applications are different. A flow battery can use relatively expensive separators, because the separator is a small component of the total flow-battery system cost (that includes pumps, automated controls and storage tanks). In EDLCs, the separators must be inexpensive because they are identical in size to the large-area-activated carbon electrodes. The redox couples in the catholyte and anolyte must also be stable together, as it would be practically challenging in a redox EDLC to prevent crossover by sealing the catholyte from the anolyte compartments. Redox EDLCs require careful control and optimization of the carbon porosity, as the carbon serves as the reservoir for the redox electrolyte and also contributes substantial capacitive energy storage capability. Practically, flow batteries find application in grid-scale energy storage, whereas redox EDLCs are targeted for high-power applications, for example, in transportation.

To better understand redox EDLCs and predict performance limits, we developed an electrochemical model. At every state of charge, the capacitive and faradaic charge passed to (or from) one electrode is a function of the electrode potential *E*_el_. The capacitive contribution to the charge *q*_cap_ is


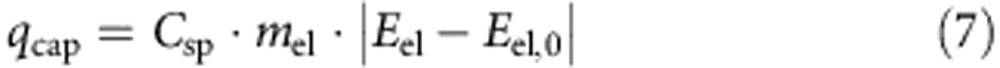


where *C*_sp_ is the specific electrode capacitance, *m*_el_ is the electrode mass and *E*_el,0_ is the electrode potential in the discharged state. The faradaic contribution is derived from the Nernst equation (that is, assuming fast electrode kinetics).


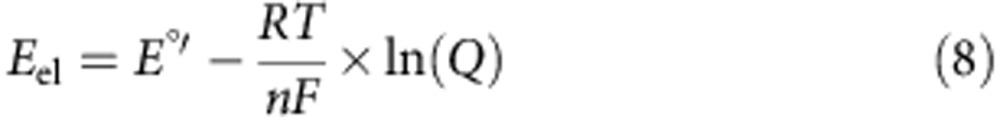


*E*_el_ is the electrode potential, *R* is the gas constant, *T* is the temperature in K, *n* is the number of electrons in the half-reaction and *E*°′ is the formal potential. *Q* is the reaction quotient for the general redox half-reaction, *x*X+*n*e^−^→*y*Y, and is a function of the total charge transferred through redox reactions (*q*_red_), the initial redox-species concentration and the electrolyte volume. Combining equations yields *q*_red_ as a function of *E*_el_ (see Supplementary Note 4).

The total faradaic charge passed through the electrode *q*_far_ at a potential *E*_el_ is:


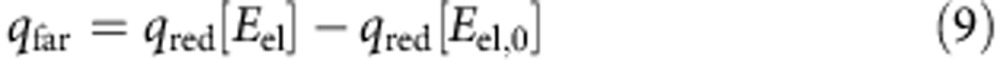


The total charge passed for an electrode is the sum of faradaic *q*_far_ and capacitive *q*_cap_ components.


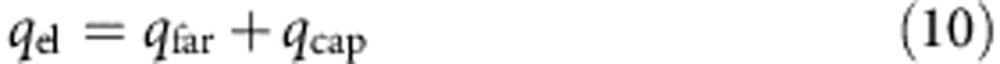


The complete cell operates under the constraints that the total charge passed (*q*_cell_) is equal to the charge passed at the positive *q*_p_ and negative *q*_n_ electrodes.


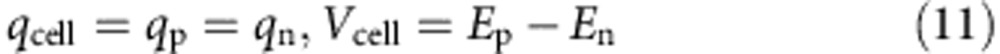


**Figure 5:**
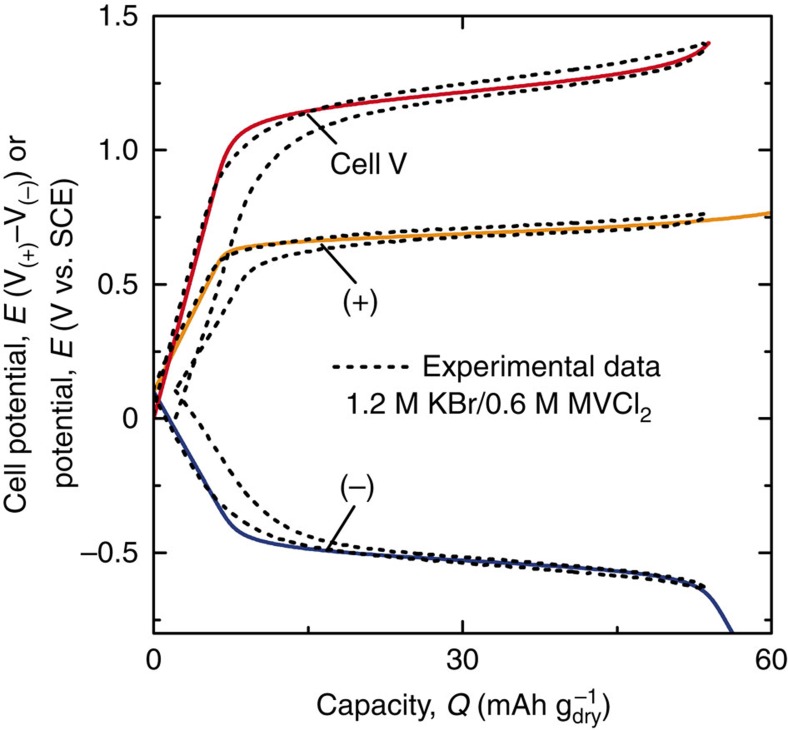
Model calculations compared with experimental data for galvanostatic charge/discharge profiles. The experimental data (dashed lines) are for a 1 M KBr/0.5 M MVCl_2_ (nominal) cell cycled to 1.4 V at 0.5 A g_dry_^−1^. The experimental charge and discharge curves are overlaid to illustrate the hysteresis and coulombic efficiency of the real cell. The model parameters that best fit the experimental data are: *C*_sp,+_=95 F g^−1^, *C*_sp,−_=105 F g^−1^, 
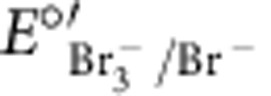
=0.70 V versus SCE, 
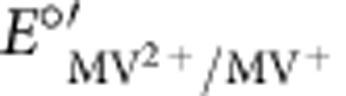
=−0.53 V versus SCE, [KBr]=1.2 M, and [MVCl_2_]=0.6 M. All other values, such as electrode mass and dimensions, electrolyte density, free volume available to electrolyte, and *E*_el,0_ were measured experimentally or calculated directly from experimental measurements.

The above set of equations are solved numerically to generate charge–discharge profiles for the redox capacitors, as are overlaid on experimental data in Fig. 5.

Comparing the model to experimental data provides insight into device operation. The formal potential of MV^2+^/MV^+^ (in the activated carbon) is found to be −0.53 V versus SCE, less negative than the standard potential of −0.69 V versus SCE^41^,^43^. From the electrochromics literature, it is known that viologen dications are reversibly reduced to form adsorbed [V^+^][X^−^] layers on the electrode at potentials less negative than the standard potential^41^,^42^,^51^,^63^,^64^. This precipitation of HV^+^ with Br^−^ is consistent with measured impedance spectra that show increased resistance after HV^+^ adsorption (Supplementary Fig. 16). A similar mechanism occurs at the positive electrode, where the formal potential for Br^−^/Br_3_^−^ from the model is 0.70 V versus SCE, less positive than the standard potential of 0.81 V versus SCE^34^. The formal potentials obtained from the model thus indicate strong adsorption at both electrodes, consistent with the separate self-discharge and adsorption measurements described earlier.

Because the electrolyte volume and total faradaic charge passed through the cell are known, it is also possible to calculate the initial concentration of redox-active species. Electrodes soaked in 1 M KBr/0.5 M MVCl_2_ before cell assembly end up with an effective MV^2+^ concentration close to 0.6 M and electrodes soaked in 1 M KBr/0.1 M MVCl_2_ or 0.4 M KBr/0.1 M HVBr_2_ end up with a viologen concentration close to 0.4 or 0.5 M. Submerging the electrodes in excess electrolyte during fabrication apparently leads to concentration of the viologen in the activated carbon due to physical adsorption, an effect observed by others^48^. In addition, the vacuum steps during electrolyte filling cause some electrolyte evaporation, increasing the concentration of all species by 18±8%.

**Figure 6:**
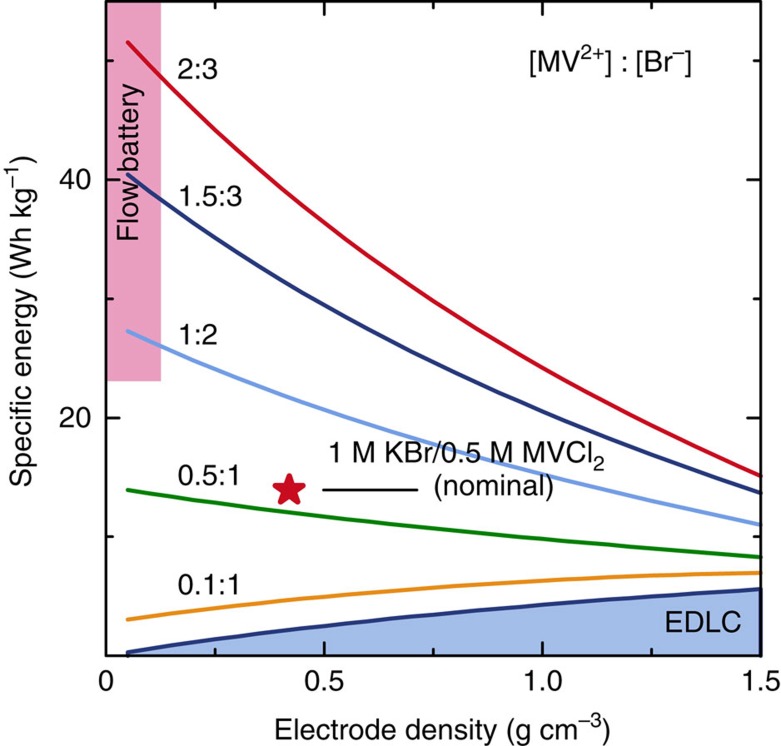
Specific energy predictions using the electrochemical model for the KBr/MVCl_2_ system. The numbers indicate different concentrations (in mol l^−1^) of redox-active species in the electrolyte for a range of activated carbon electrode densities. The specific energy is normalized to the mass of electrodes and electrolyte, and the specific capacitance of each electrode is modelled as 100 F g^−1^. For very low electrode density, the performance is determined only by the redox-active electrolyte (somewhat akin to a ‘static' flow battery). Without redox-active species the pure EDLC behaviour is recovered, as indicated by the blue region at the bottom of the plot. The experimental data for the 0.5 M MVCl_2_/1 M KBr (nominal) cell with a density of 0.42 g cm^−3^ and capacitance of 108 F g^−1^ is indicated by the red star.

The agreement between experiment and simulation indicates that the redox-EDLC operates as described by the electrochemical model and that the redox couples in the solution appear to be entirely utilized. The simulation thus enables prediction of the new technology's performance limits and potential for further improvement. By adjusting variables including electrode density, which determines the free volume available for redox-active electrolyte, and the concentration of redox-active species, we calculated the performance of different cell configurations (Fig. 6). The simulations suggest that by finding a viologen with the stability of HV and the high solubility of MV, a specific energy two to three times higher than what is reported here is experimentally possible.

In summary, we report redox EDLCs with new viologen couples and record specific energies of 14 Wh kg_wet_^−1^ (1 M KBr/0.5 M MVCl_2_) and 11 Wh kg_wet_^−1^ (0.4 M KBr/0.1 M HVBr_2_) when accounting for the complete electrolyte and electrode mass. As redox-active ions compose the electrolyte, the approach converts the ‘dead' weight of a conventional EDLC electrolyte into an active element for charge storage. The cells use neutral-pH electrolyte, can be assembled without a dry room or glove box, and have slow self-discharge similar to commercial non-aq. cells—without the use of an ion-selective membrane separator that is needed in other systems. By studying related redox electrolytes, we showed that the slow self-discharge is due to adsorption of the charged form of the redox couples (for example, Br_3_^−^ and MV^+^ or HV^+^) onto the activated carbon and that simple electrostatic effects were unable to prevent self-discharge. Our electrochemical model quantitatively describes the measurements and demonstrates that, with targeted design of new couples, the energy density could be further improved by a factor of 3 to near 50 Wh kg^−1^. These systems may thus find use in applications requiring energy and power performance in between that of batteries and traditional electrochemical capacitors.

## Methods

### Carbon electrodes

Nanoporous carbon was prepared by CO_2_ activation from high-purity carbon powder (Donacarbo, Osaka Gas Co.). Carbon precursor (1 g) positioned in the centre of a tube furnace was heated to 890 °C under flowing CO_2_ (100 ml min^−1^) for 22.5 h, which resulted in burn-off of 73.2% of the carbon mass and resulted in a Brunauer-Emmett-Teller (BET) surface area of 2,470 m^2^ g^−1^. The methylene blue-accessible surface area is 1,620 m^2^ g^−1^ (Supplementary Note 5 and Supplementary Fig. 17). The elemental composition (from inductively coupled plasma analysis) and pore size distribution of the prepared activated carbon were characterized (Supplementary Table 3 and Supplementary Fig. 18). Further details of the activated carbon preparation are reported elsewhere^65^. Activated carbon, polytetrafluoroethylene binder and acetylene black conductive additive were mechanically mixed with a 90:5:5 weight ratio. A 10-mm-diameter electrode pellet was fabricated from ∼10 mg (∼22 m^2^ per pellet) of the mixture by hydraulic pressing at 2,000 psi. To enhance the wetting of the electrode, the electrode pellets were immersed in excess electrolyte under vacuum for 10 min (to remove the air in the void space), and then pressurized with N_2_ at 150 psi to force electrolyte into the pores. The process was carried out twice to ensure the electrolyte was thoroughly infiltrated.

### Electrolytes

The electrolytes were prepared by either dissolving redox-reactive or electrochemically inert salts in 18.2 MΩ water. The salts were obtained as follows: KI (99%, Alfa Aesar), KBr (99.999%, Alfa Aesar), MVCl_2_ hydrate, MVCl_2_ (98%, Sigma Aldrich), HV dibromide, HVBr_2_ (98%, TCI AMERICA), K_2_SO_4_ (99.99%, Alfa Aesar), K_4_[Fe(CN)_6_·3H_2_O] (Mallinckrodt) and Ru(NH_3_)_6_Cl_3_ (99%, Strem Chemicals). Co(bpy)_3_Cl_2_ was prepared by literature methods^66^.

### Analytical electrochemistry

All electrochemical measurements were performed with a potentiostat/galvanostat (SP-300, Bio-logic). Redox-active electrolytes were first studied using a standard three-electrode configuration with a Pt or GC working electrode. Electrode discs were hand-polished for 30 s using 0.25 μm alumina/water slurry on Buehler microcloth. The electrode was then rinsed and sonicated in 18.2 MΩ water for 30 s. A coiled Pt wire and saturated calomel electrode (SCE, Fisher Scientific) served as the counter and reference, respectively. Test solutions (10 ml) were sparged with N_2_ for 10 min to remove dissolved O_2_.

### Three-electrode cell design

The cell was built from a perfluoroalkoxy (PFA) T-shaped Swagelok fitting and used insulated GC current collectors (Supplementary Fig. 1a). Between the activated carbon pellets, a paper separator (Whatman qualitative filter paper, Grade 1) was inserted to prevent direct electrical contact between electrodes. The electrodes/separators were soaked in test electrolyte (deaerated by flowing N_2_ gas for 10 min prior to use), and pressed in between two current-collector rods. The cell was then flooded with 0.4–0.5 ml of excess electrolyte. The rods were made of a GC plate (3-mm thick, type 2, Alfa Aesar) bonded to a Ni (Nickel Alloy 200, McMaster) body with the sides insulated with non-conducting epoxy (Stycast 1,266, Emerson and Cuming). Before each test, the GC surface was polished as with the analytical electrochemistry. The use of GC avoids potential complications due to background corrosion of, for example, stainless-steel current collectors often used in Swagelok cells. A SCE reference electrode placed with the tip at the edge of the separator was incorporated to allow for independent measurement of the absolute potential at each electrode (Supplementary Fig. 1). The cell exterior was purged with wet N_2_ during voltammetry and constant-current charge/discharge cycling.

### Cell design for specific energy and power measurements

An 11.3-mm-wide cylindrical chamber machined from inert plastic (Kel-F) served as the cell housing (Supplementary Fig. 1b). Two GC discs tightly fit with o-ring seals and back-contacted with Cu wire served as the current collectors. The separator (Polycarbonate membrane filter, STERLITECH) was cut into a disc with an identical diameter as the chamber. The electrodes were 10-mm-diameter pellets, fabricated as described above.

### Control/comparison devices

Commercial electrochemical capacitors were tested to provide reference self-discharge and performance data (Supplementary Fig. 11). One capacitor utilizes organic electrolyte with a specified 2.7 V of working potential range (BACP0010 P270 T01, Maxwell Technologies). The other (CLG03P025L12, Cellergy) utilizes aq. electrolyte, and the operating potential is specified at 3.5 V (presumably obtained using at least three cells in series).

### Reproducibility

All EDLC measurements were reproduced at least three times, with the exception of the long-term cycling studies and 24 h self-discharge tests. Representative data are shown in all cases.

## Additional information

**How to cite this article:** Chun, S-E. *et al.* Design of aqueous redox-enhanced electrochemical capacitors with high specific energies and slow self-discharge. *Nat. Commun.* 6:7818 doi: 10.1038/ncomms8818 (2015).

## Supplementary Material

Supplementary Information Supplementary Figures 1-18, Supplementary Tables 1-3, Supplementary Notes 1-5 and Supplementary References

## Figures and Tables

**Figure 1:**
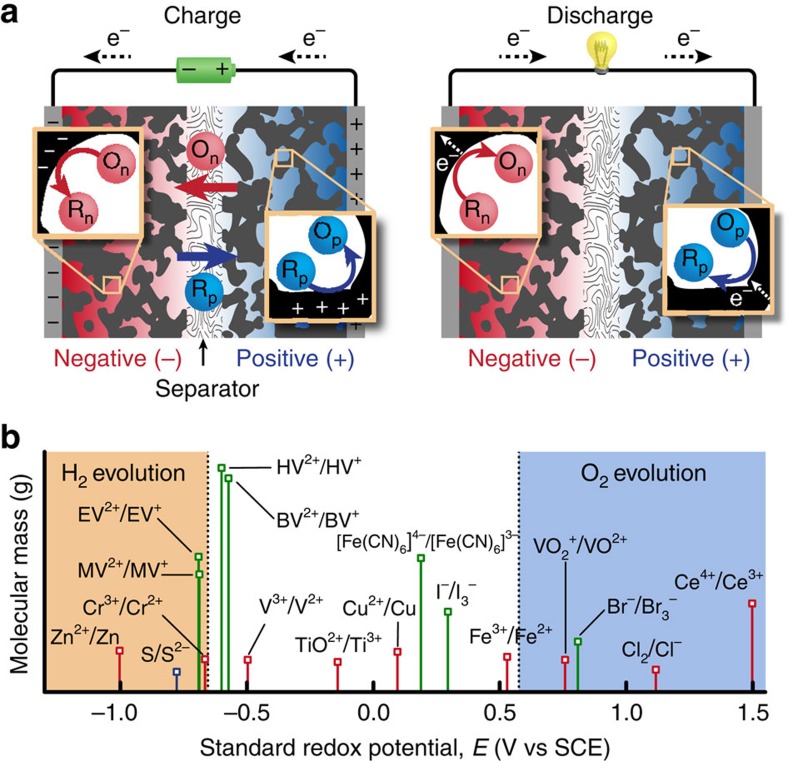
Microscopic processes and candidate couples for redox EDLCs. (**a**) Schematic showing capacitive and faradaic charge-storage processes. The redox couple used at the positive electrode (which is oxidized on charging, and reduced on discharge) is labelled as O_p_/R_p_ (catholyte), and the couple used at the negative electrode (which is reduced on charging, and oxidized on discharge) as O_n_/R_n_ (anolyte). (**b**) Reduction potentials of the couples considered relative to the thermodynamic stability window of water at neutral pH (white region). The lines coloured in red, green and blue are for couples stable in acidic (1 M acid), neutral^34^,^37^,^40^,^41^,^67^,^68^,^69^ and basic^70^ (1 M base) conditions, respectively. BV, benzyl viologen; EV, ethyl viologen; HV, heptyl viologen; MV, methyl viologen; SCE, standard calomel electrode.

**Table 1:** Performance metrics obtained from data shown in Fig. 3 collected using Swagelok cells.

**Performance metric**	**1 M KBr/0.1 M MVCl**_**2**_			**0.1 M HVBr**_**2**_		
	**1.0 V**	**1.2 V**	**1.4 V**	**0.8 V**	**1.0 V**	**1.2 V**
Specific capacity*, *Q*_dis_ (mAh g_dry_^−1^)	9.2	18.0	37.9	4.0	9.0	32.1
Specific energy*, *E*_dis_ (Wh kg_dry_^−1^)	5.2	14.5	38.7	1.3	4.8	26.4
Coulombic efficiency, *η*_C_ (0)	98.8%	99.0%	98.8%	99.9%	99.4%	99.9%
Energy efficiency, *η*_E_ (0)	99%	92%	92%	63%	80%	87%
Energy retention, *η*_R_ (6 h)	43%	33%	24%	64%	64%	86%

^*^Normalized to carbon electrode mass only

**Table 2:** Performance metrics for electrolyte-volume-limited cells.

**Redox-active species**	**1 M KBr/0.5 M MVCl_2_**	**0.4 M KBr/0.1 M HVBr_2_**
Electrode masses (mg) (including binder and conductive additive)	Cathode: 20.7 mg; Anode: 20.6 mg	Cathode: 10.0 mg; Anode: 10.0 mg
Electrolyte mass (mg)	110.1 mg	53.1 mg
*m*_electrode_/*m*_electrolyte_	1:2.69±0.02	1:2.67±0.07
Operating voltage (V)	1.4 V	1.2 V
Specific capacity*, *Q*_dis_	13.3 mAh g_wet_^−1^ (48.8 mAh g_dry_^−1^)	12.1 mAh g_wet_^−1^ (44.0 mAh g_dry_^−1^)
Specific energy*, *E*_dis_	13.9 Wh kg_wet_^−1^ (51.0 Wh kg_dry_^−1^)	10.8 Wh kg_wet_^−1^ (39.3 Wh kg_dry_^−1^)
Power density*, *P*_dis_	142 W kg_wet_^−1^ (521 W kg_dry_^−1^)	122 W kg_wet_^−1^ (447 W kg_dry_^−1^)

^*^The performance data were normalized by both electrodes and electrolyte mass and, in parenthesis, by electrode mass only.
