# Supplementary material for: Design of aqueous redox-enhanced electrochemical capacitors with high specific energies and slow self-discharge
Source: Nat Commun. 2015 Aug 4;6:7818. doi: 10.1038/ncomms8818 (PMC4532795; doi:10.1038/ncomms8818)
Supplement: Supplementary Information — Supplementary Figures 1-18, Supplementary Tables 1-3, Supplementary Notes 1-5 and Supplementary References [file ncomms8818-s1.pdf]

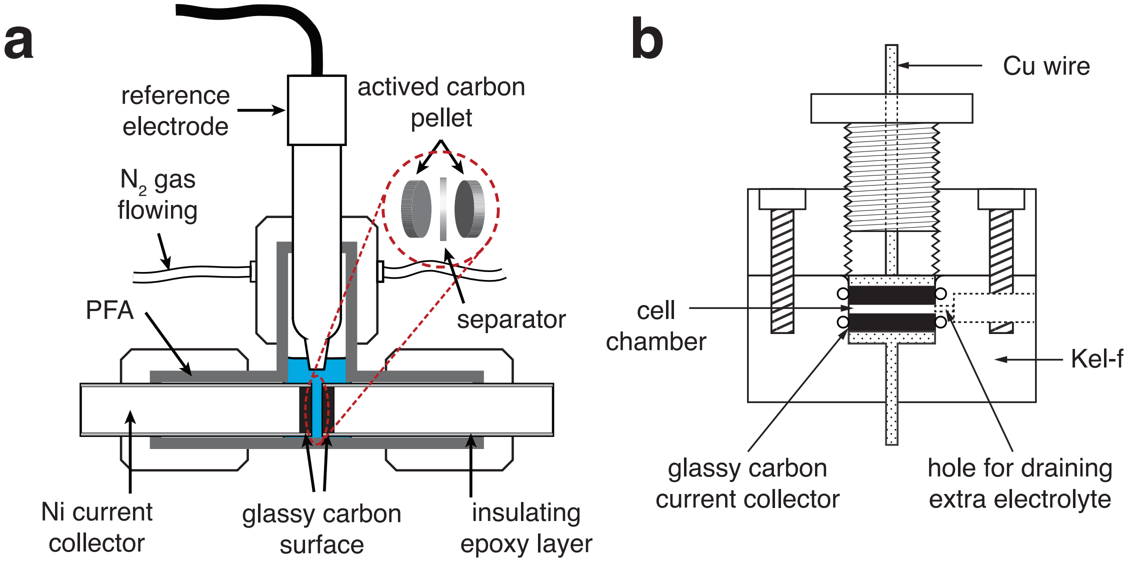

Supplementary Figure 1. Schematic of (a) the three-neck Swagelok cell and (b) custom volume-limiting cell with limited chamber volume to contain electrodes and minimal excess electrolyte

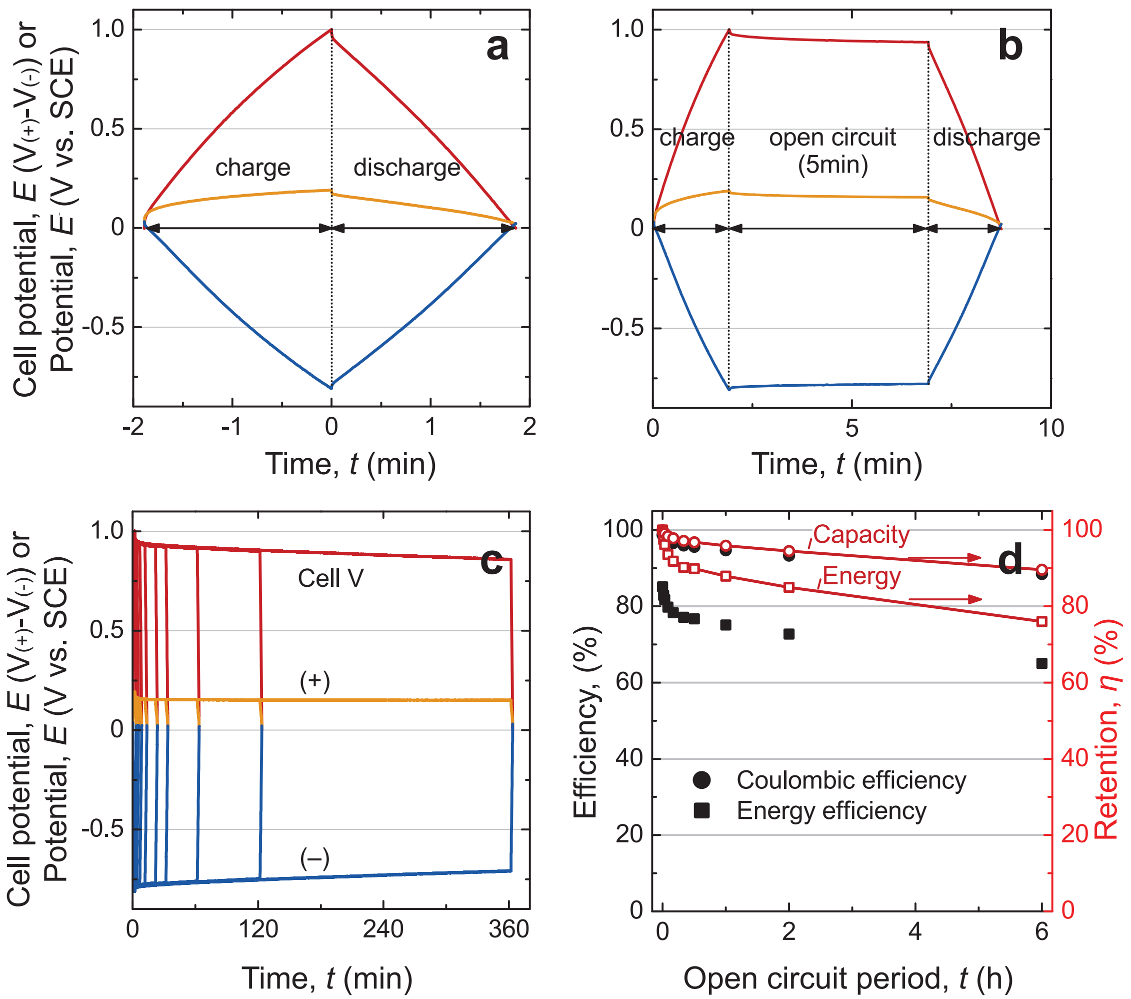

Supplementary Figure 2. Galvanostatic charge/discharge profile measured in 1 M KI solution (a) without self-discharge and (b) with 5 min self-discharge. (c) The collected galvanostatic cycling data measured at various self-discharge periods, and (d) the corresponding absolute coulombic and energy efficiencies and relative retention of these quantities.

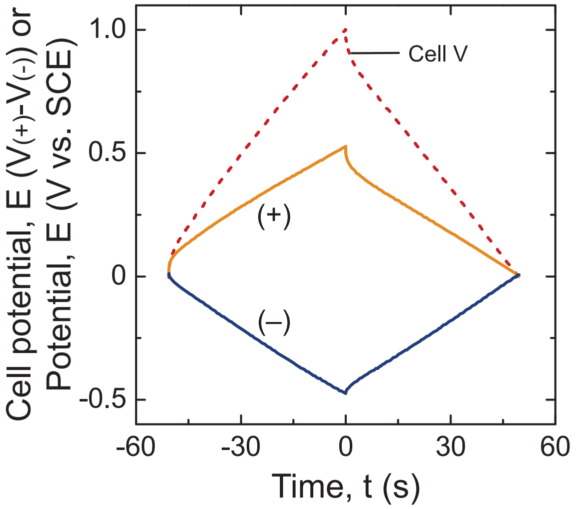

Supplementary Figure 3. Galvanostatic charge/discharge profile of symmetric two-electrode cell (1:1 mass ratio) with 1 M KBr electrolyte collected at 0.5 A g_{dry}^{-1} current density. For the symmetric two-electrode cell, the potentials of each electrode deviate symmetrically away from the initial rest potential during charging. When the cell is operated to 1 V, the positive electrode is polarized to 0.5 V vs. saturated calomel electrode (SCE) with linearly increasing potential indicating electrostatic double-layer charging (Supplementary Fig. 11). In other words, the positive potential is not sufficiently driven to oxidize Br^- to Br_3^-(which has a standard potential of 0.81 V vs. SCE).

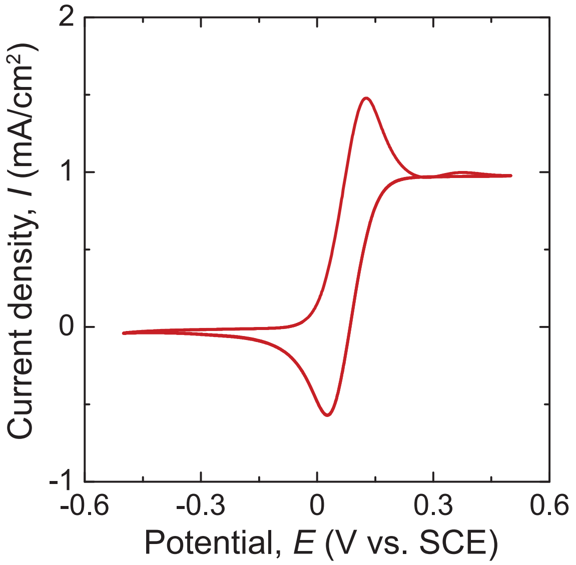

Supplementary Figure 4. Cyclic voltammogram of 0.1 M $Co(bpy)_3Cl_2$ solution measured at 5 mV s^{-1}. A three-electrode half-cell configuration was used, with Pt disc working, coiled Pt counter, and SCE reference electrode. The redox potential of $Co(bpy)_3^{2+}/Co(bpy)_3^{3+}$ was measured to be ~0.08 V vs. SCE.

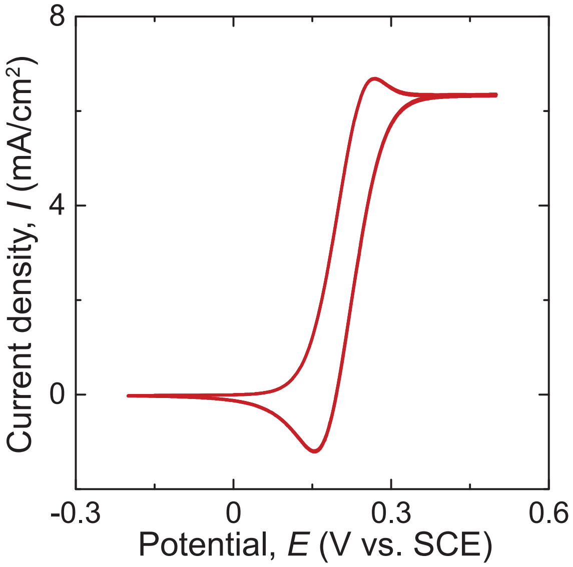

Supplementary Figure 5. Cyclic voltammogram of 0.1 M $K_4Fe(CN)_6$ solution collected at 5 mV s^{-1} scan rate. A three-electrode half-cell configuration was used, with platinum disc working electrode, coiled platinum counter electrode and saturated calomel reference electrode. The redox potential between $Fe(CN)_6^{4-}/Fe(CN)_6^{3-}$ was measured to 0.21 V (vs. SCE)

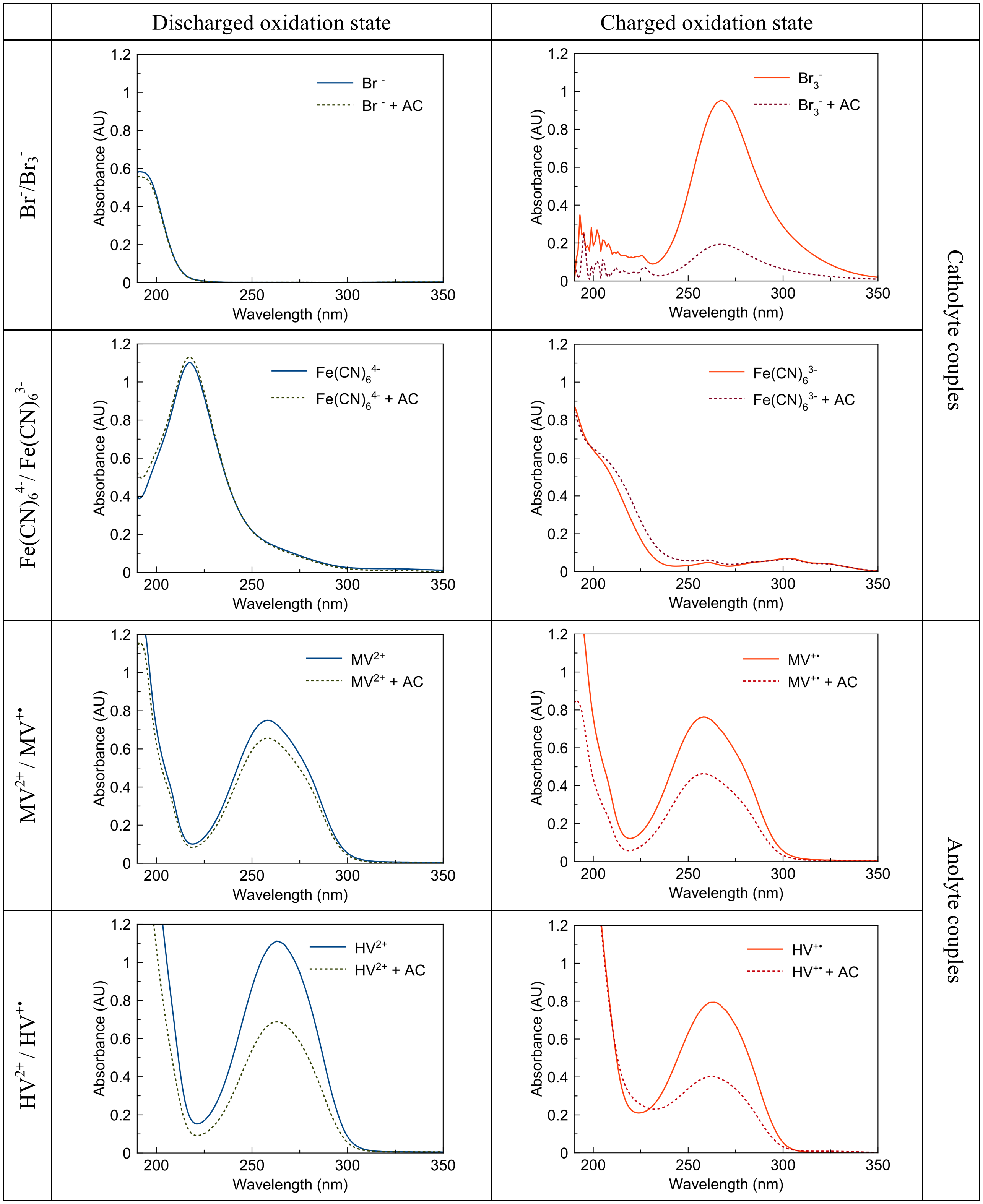

Supplementary Figure 6. UV-VIS spectra for redox couples in both charged and discharged oxidation states, with and without the addition of activated carbon.

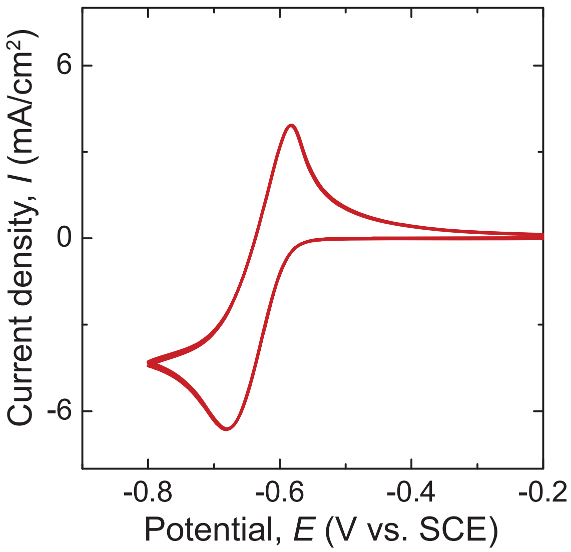

Supplementary Figure 7. Cyclic voltammogram of 0.1 M $MVCl_2$ solution measured at 5 mV s^{-1} scan rate. A three-electrode half-cell configuration was used, with Pt disc working, coiled Pt counter, and SCE reference electrode. The redox potential between MV^{2+}/MV^{+} was estimated to be -0.63 V vs. SCE with this measurement.

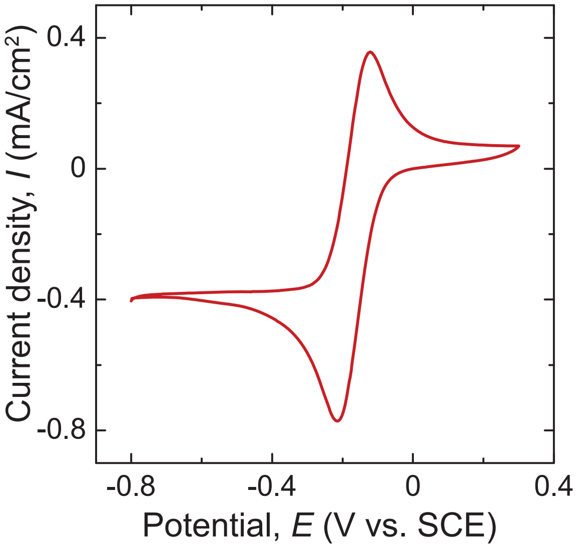

Supplementary Figure 8. Cyclic voltammogram of 0.01 M hexaammineruthenium (III) chloride ($Ru(NH_3)_6Cl_3$) solution measured at 5 mV s^{-1}. A three-electrode half-cell configuration was used, with Pt disc working, coiled Pt counter, and SCE reference electrode. The reduction potential of $Ru(NH_3)_6^{3+}/Ru(NH_3)_6^{2+}$ was estimated to be -0.17 V vs. SCE with this measurement.

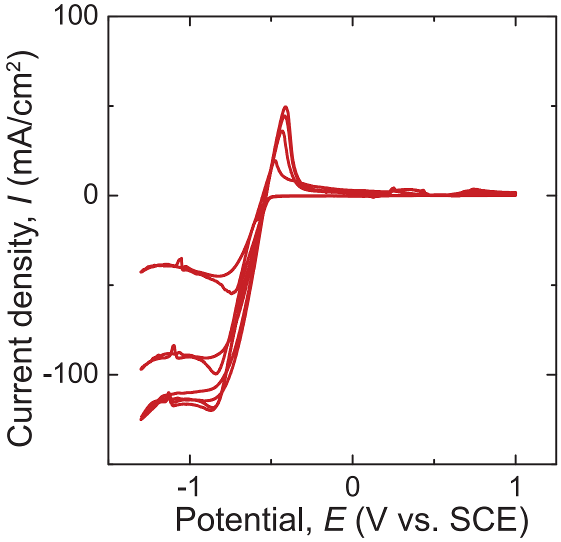

Supplementary Figure 9. Cyclic voltammogram of the mixed 0.3 M KI/0.3 $MVCl_2$ solution collected at 5 mV s^{-1}. A three-electrode half-cell configuration was used, with GC disc working electrode, coiled Pt counter electrode and SCE reference electrode. In cathodic scans, the reduction peak of MV^{2+} was observed near -0.7 V in the CV. Simultaneously, a black spike-shaped solid grew on the GC surface identified as irreversible $MV^{\bullet+}$-I^- formation.[1] Moreover, the redox reaction of I^-/I_3^- was not observed.

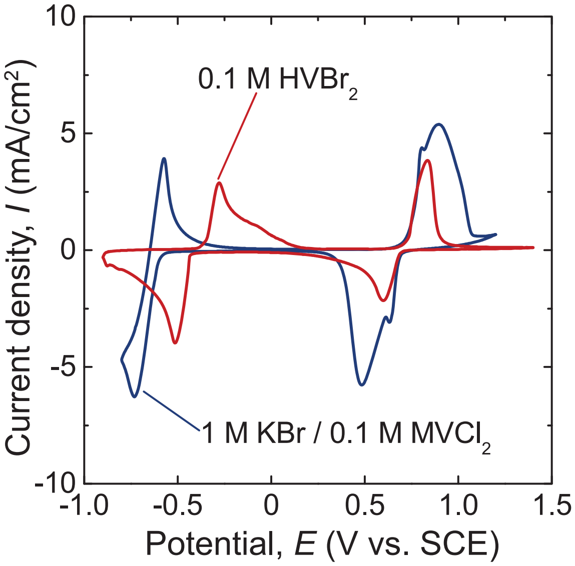

Supplementary Figure 10. Cyclic voltammogram of the mixed solution of 1 M KBr/0.1 M $MVCl_2$ and 0.1 M $HVBr_2$ collected at 5 mV s^{-1}. A three-electrode half-cell configuration was used, with GC disc working, coiled Pt counter and SCE reference electrode. Both solutions show the redox process between Br^-/Br_3^- near 0.72 V vs. SCE. Other redox processes from MV^{2+}/MV^+ at -0.64 V and HV^{2+}/HV^+ at -0.40 V vs. SCE are observed in 1 M KBr/0.1 $MVCl_2$ and 0.1 $HVBr_2$ solution, respectively. Both solutions show reversible redox behavior for both redox couples, without irreversible interaction between them.

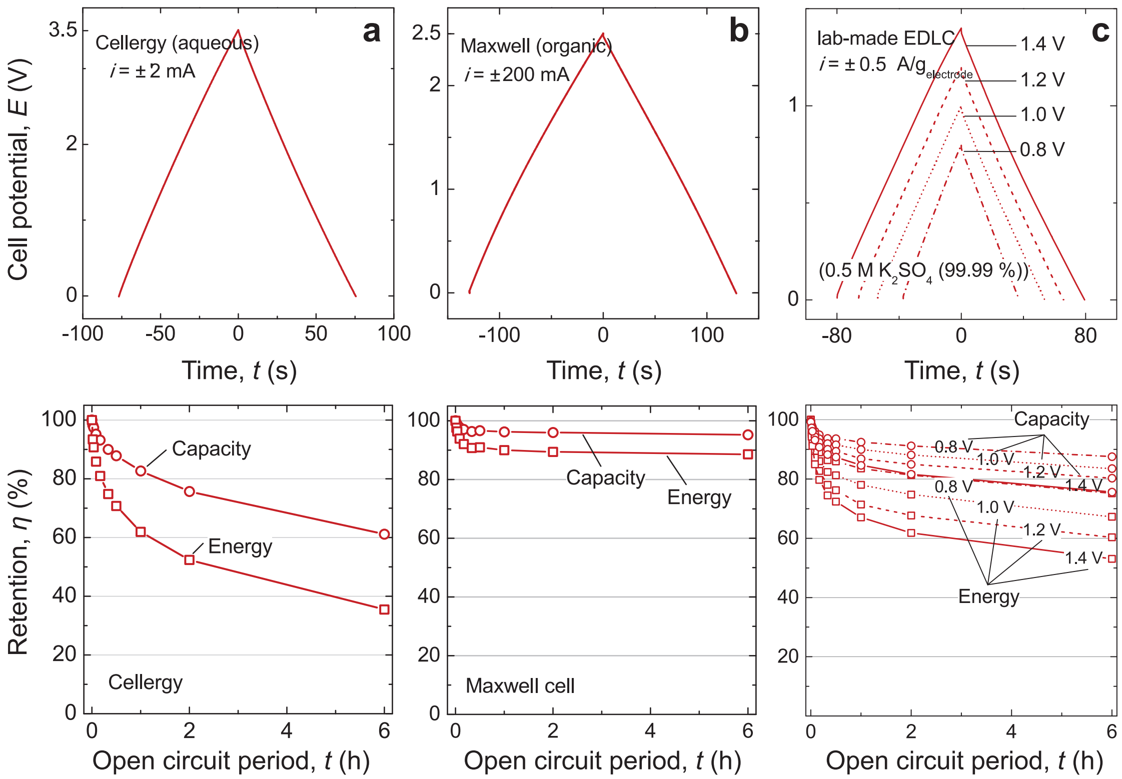

Supplementary Figure 11. Galvanostatic charge/discharge profile and the corresponding self-discharge data for (a) aqueous-based commercial supercapacitor (Cellergy, CLG03P025L12), (b) organic-based commercial supercapacitor (Maxwell, BCAP0010 P270 T01), and (c) lab-made EDLC composed of CO_2 activated carbon electrodes and 0.5 M K_2SO_4 (99.99 % pure) solution.

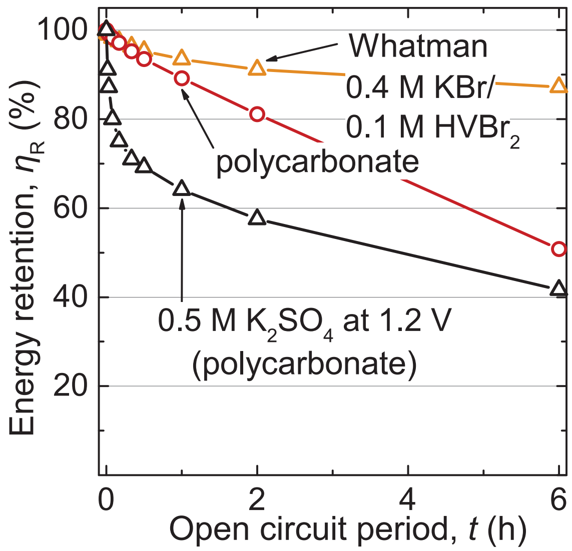

Supplementary Figure 12. Comparison of self-discharge with different separators in the 0.4 M KBr/0.1 M $HVBr_2$ cell. The self-discharge rates were collected from a volume-limiting 0.4 M KBr/0.1 M $HVBr_2$ cell with Whatman paper or polycarbonate separator.

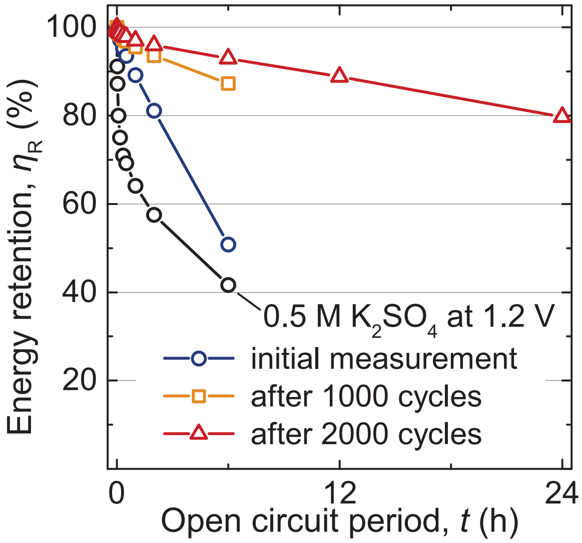

Supplementary Figure 13. The self-discharge of 0.4 M KBr/0.1 M $HVBr_2$ with polycarbonate separator measured in the volume-limiting cell, as well as that of the 0.5 M K_2SO_4 cell with the same configuration. The self-discharge was measured before long-cycling tests (0.5 A g_{dry}^{-1}), and after 1000 and 2000 cycles.

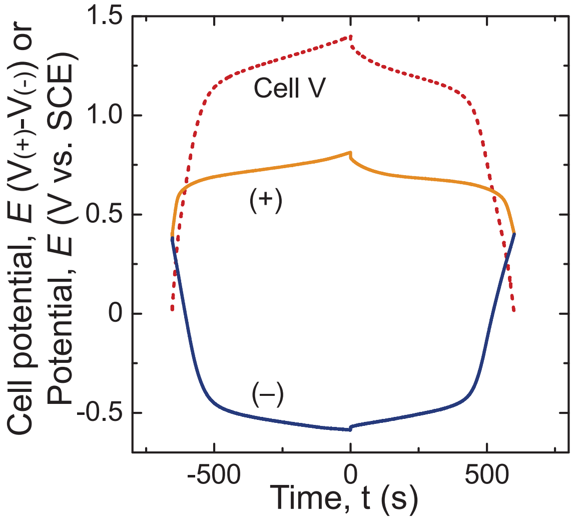

Supplementary Figure 14. Galvanostatic cycling of 1 M KBr/1 M $MVBr_2$ cell at 0.5 A g_{dry}^{-1} in a three-electrode Swagelok cell. The total cell potential (red), positive electrode potential (orange), and negative electrode potential (blue) were monitored against the SCE reference electrode. The cell cycle resulted in 92 % coulombic efficiency ($\eta_C(0)$ = 92 %).

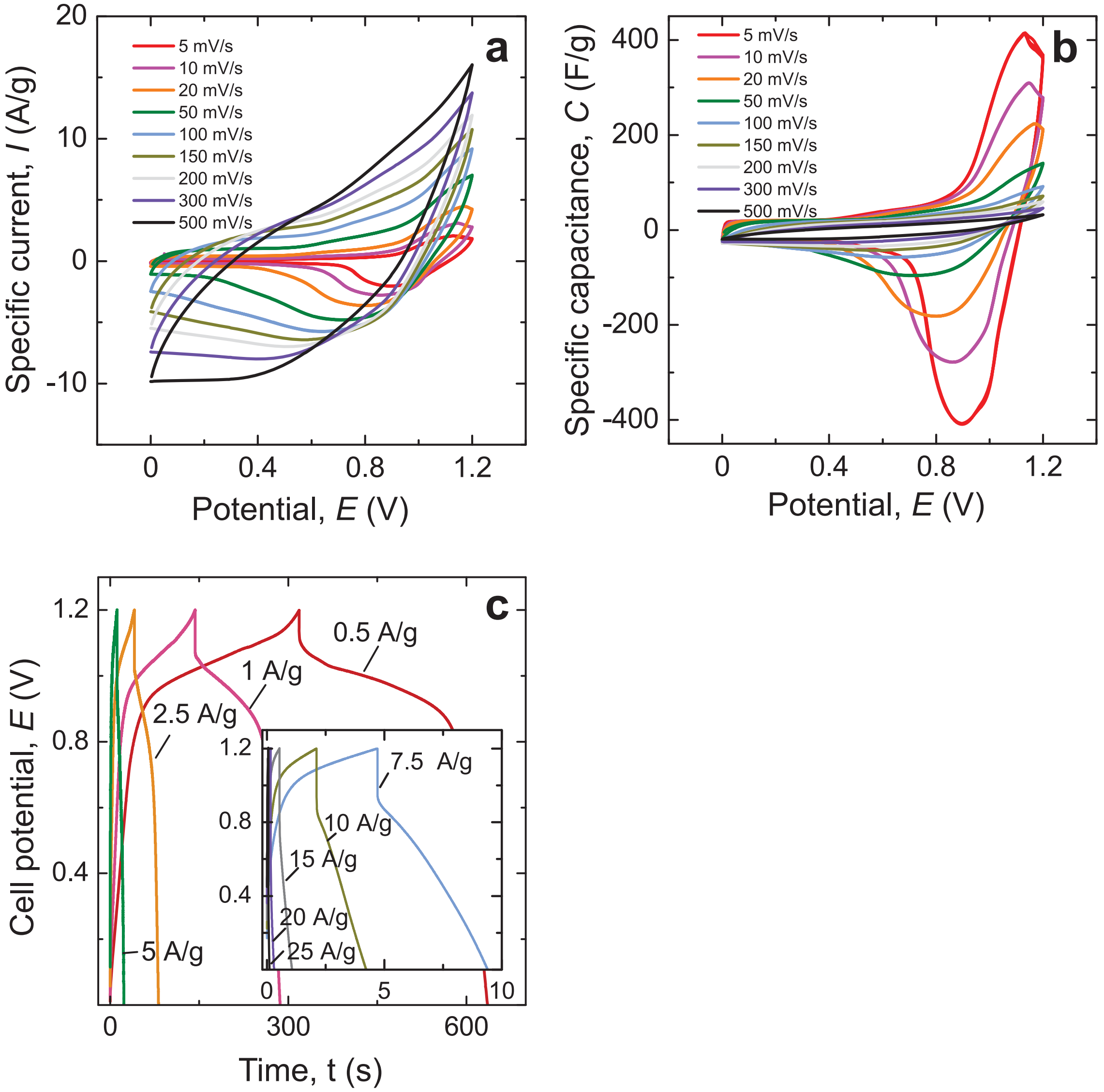

Supplementary Figure 15. (a), (b) Cyclic voltammograms at various scan rates (5 – 500 mV s^{-1}), and (c) galvanostatic charge/discharge profiles at multiple current rates (0.5 – 2.5 A g_{dry}^{-1}) measured for a 0.4 M KBr/0.1 M $HVBr_2$ cell in a two-electrode volume-limiting configuration.

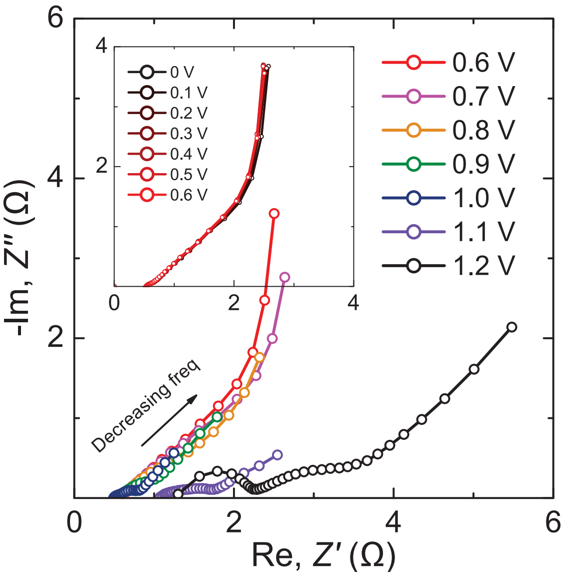

Supplementary Figure 16. Nyquist impedance spectra of the 0.4 M KBr/0.1 M $HVBr_2$ volume-limiting cell as a function of applied potential in the frequency range of 1 MHz - 0.1 Hz. Impedance spectra were measured for the 0.4 M KBr/0.1 M $HVBr_2$ cell at multiple potentials during charging/discharging. At lower operating potentials (0-0.6 V), the impedance spectra look like those of a traditional EDLC. With increasing operating potential above ~1 V, the faradic reaction with the Br^-/HV^{2+} becomes evident with the formation of a semi-circle associated with a small charge-transfer resistance. As the cell becomes completely charged at 1.2 V the "knee" in the Nyquist plot (i.e. the right side of the first semicircle) moves to higher impedance indicating an increase in the charge transfer resistance relative to that at 1 or 1.1 V (due to consumption of available redox couple which adsorbs on the carbon surface after charging). The intercept with the real axis at high frequency also increased above 1 V, indicating higher series resistance associated with the absorbed redox couple. These impedance spectra are thus consistent with HVBr precipitation on the carbon electrode.

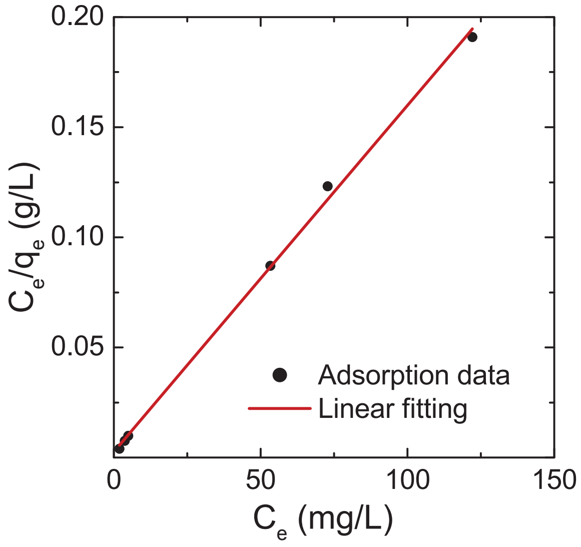

Supplementary Figure 17. Methylene blue adsorption isotherm measured from Dona activated carbon. The linear fit gives a slope of 0.001572 g mg^{-1} ($1/Q_o$), an intercept of 0.002668 g L^{-1} ($1/(Q_o*b)$), and a correlation coefficient of $R^2 = 0.998$. This gives b = 0.589 mg^{-1} and the theoretical maximum MB adsorption Q_0 = 636 mg g^{-1}. Assuming 1.35 nm^2 per MB molecule, the sample has a MB-accessible specific surface area (SSA) of 1620 m^2 g, somewhat lower than the BET surface area of 2470 m^2 g^{-1} [2].

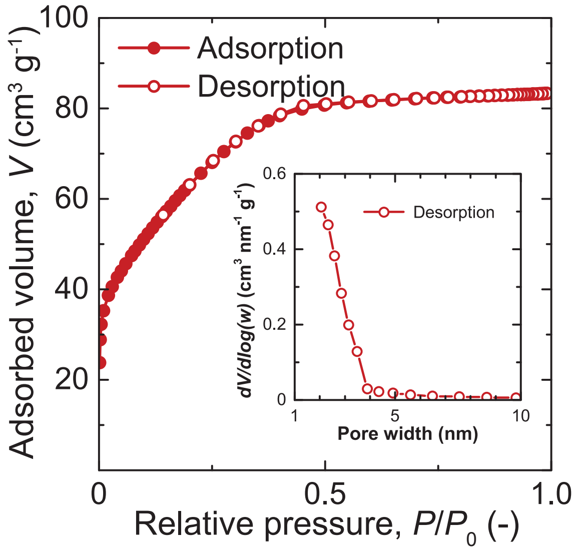

Supplementary Figure 18. N_2 gas isotherm (adsorption/desorption) graph measured at 77 K and (inset) the pore size distribution (PSD) curved based on BJH (Barrett-Joyner-Halenda) theory from the desorption isotherm.

Supplementary Table 1. Comparison of the relevant previous works including redox-EDLCs, pseudocapacitors and flow batteries

Classification	Electrode (activation agent) / dimension	Redox couple	Electrolyte	Separator	Voltage	Coulombic efficiency	Energy efficiency	E_{dry}	E_{wet}	P_{dry}	P_{wet}	Long-cycling	Self-discharge	Ref.	Notes
Redox-EDLC (single redox)	AC (KOH) / area:1.13 cm^2, *l*:N/A, m:0.030 g	Q/HQ	aq. 0.38 M hydroquinone / 1 M H_2SO_4	glassy fiber	1 V	92 %*	N.M.	31.3 Wh kg^{-1}	N.M.	24 W $kg^{-1*,a}$	N.M.	35 % (4,000 cycles) 49 %* (1,000 cycles)	N.M.	3	• aqueous electrolyte (acid)
Redox-EDLC (single redox)	AC (NaOH) / area:1 cm^2, *l*:N/A, m:0.00072 g	VO^{2+}/VO_2^+	0.3 g $VOSO_4$ / 1 M H_2SO_4	polypropylene	0.8 V	77 %	N.M.	14 Wh kg^{-1a}	N.M.	325 W kg^{-1a}	N.M.	98 % (4000 cycles)	N.M.	4	• aqueous electrolyte (acid)
Redox-EDLC (single redox)	commercial AC / N.M.	p-phenylenediamine /p-phenylenediimine	0.050 g p-phenylenediamine (PPD) / 2 M KOH	Not reported	1 V	78 %*	N.M.	19.9 Wh kg^{-1}	N.M.	17.0 kW kg^{-1} (impedance matched power) 500 W kg^{-1}*	N.M.	95 % (4000 cycles)	1 V → 0.397 V (6 h)*	5	• aqueous electrolyte (alkaline)<br>• average power is a lot less than impedance matched power
Redox-EDLC (single redox)	carbon nanotube / area:1 cm^2, *l*:280 µm, m:0.0004 g	DmFc/$DmFc^+$	0.2 M decamethylferrocenium (DmFc) / 1 M TBAP / THF	poly(tetrafluoro ethylene)	2.0 V	83 %*	N.M.	27 Wh kg^{-1}	N.M.	2.56 kW kg^{-1}	N.M.	88.4 % (10,000 cycles)	N.M.	6	• organic electrolyte<br>• high power is possibly due to the very low density of CNT on carbon paper (0.014 g/cm^3)
Redox-EDLC (single redox)	AC (KOH) / area:1 cm^2 , *l*:N.M., m:0.00072 g	Q/HQ	Hydroquinone (HQ) / PVA/H_2SO_4 gel	none	0.8 V	84%	N.M.	20 Wh kg^{-1a}	N.M.	330 W kg^{-1a}	N.M.	83 % (400 cycles)	N.M.	7	• polymer electrolyte
Redox-EDLC (single redox)	AC ($ZnCl_2$) / area:1 cm^2 , *l*:N.M., m:0.00072 g	I^-/I_3^-	0.08 M KI / 1 M H_2SO_4	polypropylene	1 V	84 %*	N.M.	19.04 Wh kg^{-1a}	N.M.	224.43 W kg^{-1a}	N.M.	No degradation (4000 cycles)	0.8 → 0.29 V (1 h)	8	• aqueous electrolyte (acid)
		Br^-/Br_3^-	0.08 M KBr / 1 M H_2SO_4	polypropylene	1 V	89 %*	N.M.	11.6 Wh kg^{-1a}	N.M.	556.8 W kg^{-1a}	N.M.	N.M.	N.M.		• aqueous electrolyte (acid)
		I^-/I_3^-	0.08 M KI / 1 M Na_2SO_4	polypropylene	1 V	91 %*	N.M.	12.3 Wh kg^{-1a}	N.M.	273.3 W kg^{-1a}	N.M.	N.M.	N.M.		• aqueous electrolyte (neutral)
Redox-EDLC (single redox)	porous carbon microspheres / area:6 cm^2, *l*:211 µm, m:0.00271 g + carbon fiber mass	Cu^{2+}/Cu	0.06 M $CuCl_2$ / 1 M HNO_3	porous plastic	1.35 V	~99 %*	N.M.	73 Wh kg^{-1a} (0.82 mWh cm^{-3})	N.M.	7.5 kW kg^{-1a} (85 mW cm^{-3})	N.M.	99.1 % (1,000 cycles)	N.M.	9	• aqueous electrolyte (acid)<br>• complex manufacturing process for electrode material<br>• low volumetric energy density due to the very low electrode density (0.023 g/cm^3)
Redox-EDLC (single redox)	graphene hydrogel / area:0.50 cm^2, *l*:440 µm, m:0.0013-0.00175 g	Q/HQ	0.4 M hydroquinone / 1 M H_2SO_4	ion-selective (Nafion 117)	0.8 V	75 %*	N.M.	8.9 Wh kg^{-1}*	N.M.	252.3 W kg^{-1}*	N.M.	80 % (1,000 cycles)	62 %* of initial energy (1 h)	10	• aqueous electrolyte (acid)<br>• expensive ion-selective membrane
Redox-EDLC (single redox)		Cu^{2+}/Cu	0.4 M $CuSO_4$ / 1 M H_2SO_4	Porous cellulose acetate	0.8 V	76 %*	N.M.	6.7 Wh kg^{-1}*	N.M.	360.0 W kg^{-1}*	N.M.	~99 % (1,000 cycles)	49 %* of initial energy (1 h)		• aqueous electrolyte (acid)
Redox-EDLC (two redox)	AC (KOH) / area:0.80 cm^3, *l*:N.M., m:0.007-0.010 g[11]	I^-/I_3^- VO^{2+}/VO_2^+	1 M KI / 1 M $VOSO_4$	glassy paper / ion-selective (Nafion 117)	0.8 V	~ 100 %	N.M.	20 Wh kg^{-1}	N.M.	51 W kg^{-1}*	N.M.	93 % (5,000 cycles)[12]	N.M.	13	• aqueous electrolyte (neutral)<br>• two-redox couples<br>• expensive ion-selective membrane
Redox-EDLC (two redox)	AC (CO_2) / area:0.79 cm^3, *l*:303 µm, m:0.010 g	Br^-/Br_3^- HV^{2+}/HV^+	0.4 M KBr / 0.1 M $HVBr_2$	polycarbonate	1.2 V	99%	82%	39.3 Wh kg^{-1} (12.7 mWh cm^{-3})	10.8 Wh/kg	447 W kg^{-1} (73.5 mW cm^{-3})	122 W kg^{-1}	99 % (2,000 cycles)	93 % of initial energy (6 h)	**This work**	• aqueous electrolyte (neutral)<br>• two-redox couples
		Br^-/Br_3^- MV^{2+}/MV^+	1 M KBr / 0.5 M $MVCl_2$	polycarbonate	1.4 V	94%	92 %	51.0 Wh kg^{-1} (16.8 mWh cm^{-3})	13.9 Wh/kg	521 W kg^{-1} (171.8 mW cm^{-3})	142 W kg^{-1}	30 % (300 cycles)	47 % of initial energy (1 h)	**This work**	• aqueous electrolyte (neutral)<br>• two-redox couples
Flow battery	carbon paper / area:2 cm^2, *l*:7.5 µm, m:N.M.	Br^-/Br_3^- AQDS/$AQDSH_2$	(+) : 0.5 M Br_2 / 3 M HBr (-) : 1 M AQDS / 1 M H_2SO_4	ion-selective (Nafion 212)	1.5 V	> 99 %	N.M.	N/A	N/A	> 0.6 W cm^{-2}	N/A	N.M.	N.M.	14	• aqueous electrolyte (acid)
Pseudo-capacitor	composite (AC+organic) / area:0.38 cm^2, *l*:N.M., m:0.0004 g (+) : AC + TCHQ (-) : AC + DCAQ	TCQ/TCHQ DCAQ/$DCAQH_2$	0.5 M H_2SO_4	Not reported	1 V	99%	Not reported	15 Wh kg^{-1}*	N.M.	63 W kg^{-1}*	N.M.	No degradation (10,000 cycles)	N.M.	15	• aqueous electrolyte (acid)

* The values are estimated based on the provided figures, but not explicitly reported in the paper

[a]metrics normalized to only active material mass except binder and conductive material mass, **N.M.** = Not measured, **N/A** = Not available, AC = activated carbon, *l*: thickness

Supplementary Table 2. The specific capacity and energy density of the lab-made control EDLC (0.5 M K_2SO_4, 99.99 % pure) normalized by combined mass of electrodes and electrolyte at different operating potentials. The values indicated as g_{dry} are normalized to the combined "dry" electrode mass. The values referred to as g_{wet} are based on the mass of both electrodes and electrolyte.

Operating voltage	0.5 M K_2SO_4 electrolyte (99.99 % pure)			
	0.8 V	1.0 V	1.2 V	1.4 V
Specific capacity	1.4 mAh g_{wet}^{-1} (5.2 mAh g_{dry}^{-1})	2.0 mAh g_{wet}^{-1} (7.5 mAh g_{dry}^{-1})	2.4 mAh g_{wet}^{-1} (9.1 mAh g_{dry}^{-1})	2.9 mAh g_{wet}^{-1} (11.0 mAh g_{dry}^{-1})
Energy density	0.5 Wh kg_{wet}^{-1} (2 Wh kg_{dry}^{-1})	0.9 Wh kg_{wet}^{-1} (3.5 Wh kg_{dry}^{-1})	1.4 Wh kg_{wet}^{-1} (5.1 Wh kg_{dry}^{-1})	1.9 Wh kg_{wet}^{-1} (7.1 Wh kg_{dry}^{-1})

Supplementary Table 3. Inductively coupled plasma (ICP) elemental analysis result on CO_2 activated Donacarbo. Only elements with over 1/1000 wt.% were shown.

CO_2 activated Donacarbo				
Al	S	Ca	Fe	Zn
0.001 wt.%	0.001 wt.%	0.004 wt.%	0.009 wt.%	0.001 wt.%

Supplementary Note 1. Details of obtaining energy retention profile as a function of self-discharge period

The self-discharge rate was studied based on the decay of either coulombic or energy efficiency as a function of open circuit time. Here, the detailed procedure of obtaining self-discharge rate is explained for one example cell composed of two symmetric electrodes and 1 M KI solution. First, the cell was charged to the specific potential by applying a constant current (0.5 A g_{dry}^{-1}). Then, while monitoring the cell potential decay, the cell was left at various periods of open circuit: 0 min, 1 min, 2 min, 5 min, 10 min, 20 min, 30 min, 60 min, 120 min, and 360 min (Supplementary Fig. 2b). After each open circuit period, the cell was discharged to 0 V by extracting the same constant current density (-0.5 A g_{dry}^{-1}). Supplementary Figure 2c plots the collected voltage profiles of each test with different self-discharge times. For each self-discharge test, at each time, the coulombic and energy efficiency (%) were obtained.

Supplementary Note 2. UV-VIS absorption spectroscopy of Br^-/Br_3^-, $Fe(CN)_6^{4-}/Fe(CN)_6^{3-}$, MV^{2+}/MV^+, and HV^{2+}/HV^+ solution

Activated Carbon adsorption experiments

In these experiments, concentrations of oxidized and reduced redox couples in an electrolyte were measured with and without the presence of activated carbon (AC). All experiments were performed in 1.5 mL centrifuge tubes. The tubes were filled with 1.5 mL of ~10 mM analyte solution and capped to exclude oxygen immediately after adding reagents and/or 5 mg of AC. A vortex mixer was used to agitate the centrifuge tubes at the beginning and end of a 10 minute waiting period. After this time, the tubes were centrifuged and a 10 uL sample of the supernatant was diluted by a factor of 200 into 1.99 mL of Millipore 18.2 MΩ.cm water in a quartz cuvette. The cuvette was shaken vigorously for 20 s to ensure a homogeneous analyte. The concentration of each analyte was determined with an Agilent UV-Vis spectrophotometer.

Methyl Viologen adsorption

For MV^{2+}, the analyte was prepared by directly dissolving $MVCl_2$ salt into 18.2 MΩ.cm water. For the MV^+ analyte, chemical reduction of the MV^{2+} solution was performed in the centrifuge tubes with excess zinc powder (8 um) with and without AC. In the cuvette, direct measurement of dilute MV^+ is difficult due to background reaction with dissolved oxygen, so the reduced viologen in the diluted sample was allowed to completely reoxidize and the concentration of the resulting MV^{2+} was measured instead.

To ensure there was no effect due to reaction time, all spectra were repeated after 20 min. Very little deviation was observed between 10 and 20 min intervals:

Sample	MV^{2+} concentration (mM)	Absorption, $A_{258\ nm}$ (AU)
$MVCl_2$	9.65	0.75024
$MVCl_2$ + Zn	9.94	0.77259
$MVCl_2$ + AC	8.44	0.65625
$MVCl_2$ + AC + Zn	5.84	0.45377

Heptyl Viologen adsorption

The adsorption of heptyl viologen ($HVBr_2$) was investigated using the same procedures as with MV. Because zinc powder did not perform well as a reducing agent for HV, 15 mg sodium dithionite ($Na_2S_2O_4$) was used instead.[16] It should be noted that even without activated carbon, some of the reduced HV^+ precipitates, suggesting the HV^+ radical is much less soluble than the MV^+ radical.

Sample	HV^{2+} concentration (mM)	Absorption, $A_{264\ nm}$ (AU)
$HVBr_2$	9.47	1.1144
$HVBr_2$ + AC	5.76	0.6779
$HVBr_2$ + $Na_2S_2O_4$	6.40	0.75427
$HVBr_2$ + $Na_2S_2O_4$ + AC	2.98	0.35076

Bromide and Bromine/Tribromide adsorption

For Br^-, the analyte was prepared by directly dissolving KBr salt into 18.2 MΩ.cm water. Because Br_2 and Br_3^- are stable in air, these oxidation products can be measured directly and these species were generated *ex-situ*, unlike the viologen radicals. Liquid bromine is volatile and difficult to handle and precisely measure, so bromine and tribromide were generated using the following chemical reactions[17]:

$$NaBrO_3 + 5\ Br^- + 6\ H^+ \rightarrow 3\ Br_{2(aq)} + 3\ H_2O \qquad (1)$$

$$Br_{2(aq)} + Br^- \leftrightarrow Br_3^- \quad (K_{eq} = 16.1\ M^{-1}) \qquad (2)$$

To increase the conversion of the sodium bromate and drive the equilibrium products towards Br_3^-, the oxidation was performed in a solution containing 0.1 M KBr and 0.1 M HBr with $NaBrO_3$ as the limiting reagent. This reaction, if completed, produces a total bromine concentration of 10 mM:

$$[Br_2]_T = [Br_2] + [Br_3^-] = 0.01\ M \ \ \text{(before adsorption to AC)} \qquad (3)$$

Like other species investigated by UV-vis, the supernatant from the Br^- tests was diluted by a factor of 200 in Millipore 18.2 MΩ cm water. Samples from the oxidized Br_2/Br_3^- species, however, were diluted with a 0.1 M KBr solution. This ensured that the equilibrium did not shift from Br_3^- back towards Br_2 in the cuvette, which has a much lower molar absorptivity coefficient, making it harder to accurately measure by UV-vis. The total concentration of oxidized bromide species in solution remaining, $[Br_2]_T$ is proportional to the absorption at λ_{max} = 266 nm, and can be calculated by combining equations (2) and (3) [17]:

$$A_{266} = \frac{(\epsilon_{Br_3^-})K_{eq}[Br^-][Br_2]_T}{1+K_{eq}[Br^-]} \tag{4}$$

with $\epsilon_{Br_3^-} = 40{,}900\ \mathrm{M^{-1}cm^{-1}}$

Sample	$Br_2 + Br_3^-$ concentration (mM)	Absorption, $A_{266\ nm}$ (AU)
Br_3^-	7.58	0.9557
Br_3^- + AC	1.45	0.1828

For Br^-, the analysis is simpler. No chemical equilibrium considerations are necessary and λ_{max} = 191 nm with $\epsilon_{Br^-} = 13{,}000\ \mathrm{M^{-1}cm^{-1}}$

Sample	Br^- concentration (mM)	Absorption, $A_{191\ nm}$ (AU)
Br^-	8.98	0.5836
Br^- + AC	8.74	0.5682

Ferrocyanide/Ferricyanide adsorption

For the ferrocyanide and ferricyanide analytes, no chemical oxidation or reduction was required. Instead, the 10 mM ferrocyanide solution and the 10 mM ferricyanide solution were prepared separately directly from $K_4Fe(CN)_6$ and $K_3Fe(CN)_6$ salts, respectively.

Sample	$Fe(CN)_6^{3-}$ concentration (mM)	Absorption, $A_{190\ nm}$ (AU)
$Fe(CN)_6^{3-}$	10.00	0.8762
$Fe(CN)_6^{3-}$ + AC	9.7	0.8511

Sample	$Fe(CN)_6^{4-}$ concentration (mM)	Absorption, $A_{218\ nm}$ (AU)
$Fe(CN)_6^{4-}$	10.00	1.1007
$Fe(CN)_6^{4-}$ + AC	10.3	1.1305

Supplementary Note 3. Benchmarking the commercial electric double-layer capacitors (EDLCs) and the lab-made EDLCs

Two commercial EDLCs using an aqueous and an organic electrolyte were characterized to evaluate the electrochemical behavior and self-discharge rate of commercial devices as a benchmark: organic-based EDLC (Maxwell, BCAP0010 P270 T01) and aqueous-based EDLC (Cellergy, CLG03P025L12). For an additional comparison, the lab-made EDLC cell was tested with the manufactured CO_2 activated carbon electrodes and non-redox active electrolyte (0.5 M K_2SO_4, 99.99 % pure).

Galvanostatic charge/discharge cycling was performed on each system by applying appropriate current based on the device capacity. All three kinds of EDLCs show linearly changing potential with time, indicating pure double-layer capacitive charging. For the lab-made EDLCs, the specific capacity and energy density were normalized in two ways based either on combined electrodes mass ("dry", as is typical) or on the integrated mass of electrodes and electrolyte (i.e. "wet"). The data is shown in Supplementary Table 2.

Supplementary Note 4: Electrochemical model details

Electrolyte mass is especially important for devices with redox-active electrolytes. To develop significant faradaic energy storage, a sufficient supply of redox-active ions is required. The volume of electrolyte available is determined by the free volume in the porous electrodes and the separator[18]. This volume, along with the concentration of the redox-active species, determines the maximum possible number of ions available for faradaic charging. For each electrode, the total electrode volume is as a sum of the volume of each component, as shown in equation (5):

$$V_{\mathrm{el}} = m_{\mathrm{el}}\left(\frac{f_{\mathrm{binder}}}{\rho_{\mathrm{binder}}} + \frac{f_{\mathrm{ACskeleton}}}{\rho_{\mathrm{ACskeleton}}} + \frac{f_{\mathrm{CarbonBlack}}}{\rho_{\mathrm{CarbonBlack}}}\right) + V_{\mathrm{free,el}} \qquad (5)$$

where f_x and ρ_x represent the mass fraction and density, respectively, of each component, m_{el} represents the total electrode mass, V_{el} represents the total electrode apparent volume, and $V_{\mathrm{free,el}}$ represents the free pore volume that will be filled with electrolyte upon device assembly. The solid skeleton of many activated carbons, carbon blacks, and other materials with graphitic domains has a density of approximately 2.1 g cm^{-3} and PTFE has a density of 2.2 g cm^{-3} [19-21]. The free volume of the separator is also straightforward to calculate, as illustrated in equation (6):

$$A_{\mathrm{sep}} * t_{\mathrm{sep}} * P_{\mathrm{sep}} = V_{\mathrm{free,sep}} \qquad (6)$$

Where A_{sep} and t_{sep} are the area and thickness, respectively, of the separator, P_{sep} is the separator porosity, and $V_{\mathrm{free,sep}}$ is the separator free volume. Cellulosic separators tend to have a porosity near 0.7, while

glassy fiber separators have a porosity closer to 0.9 [22]. Some popular polyethylene separators, such as Celgard 3501, have a porosity of 0.55. The separator and the electrodes are the only porous components in a complete device, so the electrolyte volume, $V_{\mathrm{electrolyte}}$, will simply be the sum of $V_{\mathrm{pore,el}}$ for both electrodes and $V_{\mathrm{free,sep}}$ as shown in equation (7):

$$V_{\mathrm{electrolyte}} = V_{\mathrm{pore,p}} + V_{\mathrm{pore,n}} + V_{\mathrm{free,sep}} \qquad (7)$$

To model the total energy storage of the system, the contributions from capacitive energy storage and faradaic energy storage are treated as separate processes occurring simultaneously. For every point in time or at every state of charge (SOC), the amount of capacitive charge and the amount of faradaic charge passed to or from an electrode can be calculated as a function of the electrode potential. The capacitive contribution for a single electrode is described in equation (8):

$$q_{\mathrm{cap}} = C_{\mathrm{sp}} * m_{\mathrm{el}} * \left|E_{\mathrm{el}} - E_{\mathrm{el,0}}\right| \qquad (8)$$

Where q_{cap} is the charge passed due to capacitive charging, C_{sp} is the specific capacitance of the electrode, m_{el} is the electrode mass, and E_{el} and $E_{\mathrm{el,0}}$ are the electrode potentials at a given voltage and in the discharged state, respectively. The faradaic contribution is derived using the Nernst equation (9):

$$E_{\mathrm{el}} = E^{0'} - \frac{RT}{nF} * \ln(Q[q_{\mathrm{red}}]) \qquad (9)$$

Here, E_{el} is the electrode potential, R is the universal gas constant, T is the temperature, n is the number of electrons involved in the electrochemical half-reaction, and Q is the reaction quotient for the anolyte or catholyte redox half-reaction. The reaction quotient is dependent upon q_{red}, the charge transferred due to redox reactions. For example, for the general redox reaction:

$$\mathrm{xX} + \mathrm{ne^-} \rightarrow \mathrm{yY} \qquad (10)$$

The reaction quotient is expressed as

$$Q = \frac{[\mathrm{Y}]^{\mathrm{y}}}{[\mathrm{X}]^{\mathrm{x}}} = \frac{\left(\frac{\frac{q_{\mathrm{red}}}{F} * \frac{\mathrm{y}}{n}}{V_{\mathrm{electrolyte}}}\right)^{\mathrm{y}}}{\left(\frac{[\mathrm{X_0}] * V_{\mathrm{electrolyte}} - \frac{q_{\mathrm{red}}}{F} * \frac{\mathrm{x}}{n}}{V_{\mathrm{electrolyte}}}\right)^{\mathrm{x}}} \qquad (11)$$

Where $[X_0]$ is the initial concentration of the anolyte or catholyte. It should be noted that no activity coefficients are included here. These are incorporated into $E^{0'}$, the experimentally determined formal potential. In a more advanced treatment, $E^{0'}$ would be dependent on the SOC instead of being treated as a constant, as the ionic strength of the electrolyte changes with time.

Combining eqns. (8) and (10) and solving gives q_{red} as a function of E_{el}. To find the total faradaic charge that has passed through the electrode at a given potential, it is necessary to use equation (12):

$$q_{\mathrm{far}} = q_{\mathrm{red}}[E_{\mathrm{el}}] - q_{\mathrm{red}}[E_{\mathrm{el,0}}] \qquad (12)$$

Finally, the total charge passed through an electrode as a function of the electrode potential is:

$$q_{\mathrm{el}}[E_{\mathrm{el}}] = q_{\mathrm{far}}[E_{\mathrm{el}}] + q_{\mathrm{cap}}[E_{\mathrm{el}}] \quad (13)$$

The complete cell must operate under the constraints that

$$V_{\mathrm{cell}} = E_{\mathrm{p}} - E_{\mathrm{n}} \quad (14)$$

$$q_{\mathrm{cell}} = q_p = q_{\mathrm{n}} \quad (15)$$

By simultaneously solving these equations using numerical methods over the range of $V_{\mathrm{cell,min}}$ to $V_{\mathrm{cell,\,max}}$, it is possible to find the total energy and capacity of the device, the charge/discharge profiles of the anode and cathode, and even the degree of conversion of each redox couple.

Supplementary Note 5: Methylene blue adsorption for surface area measurement

This test was performed using standard procedures described elsewhere.[23] Methylene blue (MB) was dissolved into Millipore 18.2 MΩ cm water. Samples of activated DonaCarbon (890ºC, 100 sccm CO_2, 22.5 h) were massed and mixed with MB solutions in 250 mL and 500 mL Erlenmeyer flasks. The mixtures were stirred at 25ºC for 24 h to reach equilibrium adsorption. Samples of the supernatant from the flasks were centrifuged to remove any activated carbon, and the concentration of MB was determined with an Agilent UV-Visible spectrophotometer at 664 nm.

The data was fit using the linear form of the Langmuir adsorption isotherm model:

$$\frac{C_e}{q_e} = \frac{1}{Q_0 * b} + \left(\frac{1}{Q_0}\right) * C_e \quad (16)$$

C_{e} = equilibrium concentration of MB in the liquid phase (mg L^{-1})

q_{e} = equilibrium concentration of MB in the solid phase (mg g^{-1})

Q_0 = theoretical maximum MB concentration in the solid phase, corresponding to the formation of a complete monolayer (mg g^{-1})

b = adsorption energy constant (mg^{-1})

Supplementary References

1. Bird C. L., Kuhn A. T. Electrochemistry of the viologens. *Chem. Soc. Rev.* **10**, 49-82 (1981).

2. Yang X., Cheng C., Wang Y., Qiu L., Li D. Liquid-mediated dense integration of graphene materials for compact capacitive energy storage. *Science* **341**, 534-537 (2013).

3. Roldán S., Blanco C., Granda M., Menéndez R., Santamaría R. Towards a further generation of high-energy carbon-based capacitors by using redox-active electrolytes. *Angew. Chem. Int. Ed.* **50**, 1699-1701 (2011).

4. Senthilkumar S. T., Selvan R. K., Ponpandian N., Melo J. S., Lee Y. S. Improved performance of electric double layer capacitor using redox additive (VO^{2+}/VO_2^{+}) aqueous electrolyte. *J. Mater. Chem. A* **1**, 7913-7919 (2013).

5. Wu J*., et al.* A simple and high-effective electrolyte mediated with p-phenylenediamine for supercapacitor. *J. Mater. Chem.* **22**, 19025-19030 (2012).

6. Park J., Kim B., Yoo Y.-E., Chung H., Kim W. Energy-density enhancement of carbon-nanotube-based supercapacitors with redox couple in organic electrolyte. *ACS Appl. Mater. Interfaces* **6**, 19499-19503 (2014).

7. Senthilkumar S. T., Selvan R. K., Ponpandian N., Melo J. S. Redox additive aqueous polymer gel electrolyte for an electric double layer capacitor. *RSC Adv.* **2**, 8937-8940 (2012).

8. Senthilkumar S. T., Selvan R. K., Lee Y. S., Melo J. S. Electric double layer capacitor and its improved specific capacitance using redox additive electrolyte. *J. Mater. Chem. A* **1**, 1086-1095 (2013).

9. Mai L.-Q*., et al.* Synergistic interaction between redox-active electrolyte and binder-free functionalized carbon for ultrahigh supercapacitor performance. *Nat. Commun.* **4**, (2013).

10. Chen L., Bai H., Huang Z., Li L. Mechanism investigation and suppression of self-discharge in active electrolyte enhanced supercapacitors. *Energy Environ. Sci.* **7**, 1750-1759 (2014).

11. Lota G., Centeno T. A., Frackowiak E., Stoeckli F. Improvement of the structural and chemical properties of a commercial activated carbon for its application in electrochemical capacitors. *Electrochim. Acta* **53**, 2210-2216 (2008).

12. Lu M., Beguin F., Frackowiak E. *Supercapacitors: Materials, Systems and Applications*. Wiley (2013).

13. Frackowiak E., Fic K., Meller M., Lota G. Electrochemistry serving people and nature: high-energy ecocapacitors based on redox-active electrolytes. *ChemSusChem* **5**, 1181-1185 (2012).

14. Huskinson B*., et al.* A metal-free organic-inorganic aqueous flow battery. *Nature* **505**, 195-198 (2014).

15. Tomai T., Mitani S., Komatsu D., Kawaguchi Y., Honma I. Metal-free aqueous redox capacitor via proton rocking-chair system in an organic-based couple. *Sci. Rep.* **4**, (2014).

16. Monk P. M. S., Hodgkinson N. M., Ramzan S. A. Spin pairing ('dimerisation') of the viologen radical cation: kinetics and equilibria. *Dyes Pigm.* **43**, 207-217 (1999).

17. Wang T. X., Kelley M. D., Cooper J. N., Beckwith R. C., Margerum D. W. Equilibrium, kinetic, and UV-spectral characteristics of aqueous bromine chloride, bromine, and chlorine species. *Inorg. Chem.* **33**, 5872-5878 (1994).

18. Merrill M. D*., et al.* Optimizing supercapacitor electrode density: achieving the energy of organic electrolytes with the power of aqueous electrolytes. *RSC Adv.* **4**, 42942-42946 (2014).

19. Purewal J. J., Kabbour H., Vajo J. J., Ahn C. C., Fultz B. Pore size distribution and supercritical hydrogen adsorption in activated carbon fibers. *Nanotechnology* **20**, 204012 (2009).

20. Zhang J*., et al.* Sustainable, heat-resistant and flame-retardant cellulose-based composite separator for high-performance lithium ion battery. *Sci. Rep.* **4**, (2014).

21. Donnet J. B. *Carbon Black: Science and Technology, Second Edition*. Taylor & Francis (1993).

22. Daniel C., Besenhard J. O. *Handbook of Battery Materials*. Wiley (2012).

23. Hameed B. H., Din A. T. M., Ahmad A. L. Adsorption of methylene blue onto bamboo-based activated carbon: Kinetics and equilibrium studies. *J. Hazard. Mater.* **141**, 819-825 (2007).
